# Allelopathic Potential of Rice and Identification of Published Allelochemicals by Cloud-Based Metabolomics Platform

**DOI:** 10.3390/metabo10060244

**Published:** 2020-06-15

**Authors:** Thi L. Ho, Tu T. C. Nguyen, Danh C. Vu, Nhu Y. Nguyen, Trang T. T. Nguyen, Trieu N. H. Phong, Cuong T. Nguyen, Chung-Ho Lin, Zhentian Lei, Lloyd W. Sumner, Vang V. Le

**Affiliations:** 1 Cuu Long Delta Rice Research Institute, Can Tho 94000, Vietnam; pnhtrieu@gmail.com (T.N.H.P.); cuongnt.clrri@mard.gov.vn (C.T.N.); 2 College of Agriculture and Applied Biosciences—Can Tho University, Can Tho 94000, Vietnam; ntct1608@gmail.com (T.T.C.N.); lvvang@ctu.edu.vn (V.V.L.); 3 Faculty of Technology, Van Lang University, Ho Chi Minh City 70000, Vietnam; dcvgwc@mail.missouri.edu; 4 Center for Agroforestry, School of Natural Resources, University of Missouri, Columbia, MO 65211, USA; nynb5b@mail.missouri.edu (N.Y.N.); linchu@missouri.edu (C.-H.L.); 5 Cultivation and Plant Protection Station. Thoi Lai District, Can Tho 94000, Vietnam; nguyenthithuytrang.jan16@gmail.com; 6 Metabolomics Center, University of Missouri, Columbia, MO 65211, USA; leiz@missouri.edu (Z.L.); sumnerlw@missouri.edu (L.W.S.); 7 Department of Biochemistry, Bond Life Sciences Center, University of Missouri, Columbia, MO 65211, USA

**Keywords:** rice, barnyardgrass, allelochemicals, metabolomics, weed control

## Abstract

The methanol extracts of nine popular cultivated Vietnamese rice cultivars (*Oryza sativa* L.cv. OM 2395, 5451, 6976, 380, 5930, 4498, 3536, N406, and 7347) were used to explore their allelopathic potential on barnyardgrass (*Echinochola crus-galli* L.). At 0.1 g mL^−1^, OM 5930, OM 4498, and OM 6976 correlatively possessed greatest phytotoxicity on barnyardgrass shoot (98.77%, 90.75%, and 87.17%) and root (99.39%, 92.83%, and 86.56%) growth. The following study aimed to detect previously-known allelochemicals in those rice using XCMS online cloud-based metabolomics platform. Twenty allelochemicals were semi-quantified and seven of them were detected predominantly and five was putatively confirmed in OM 5930 (mg/ 100g fresh rice) as salicylic acid (5.0076), vanillic acid (0.1246), *p*-coumaric acid (0.1590), 2,4-dimethoxybenzoic acid (0.1045), and cinnamic acid (3.3230). These compounds were active at concentrations greater than 0.5 mM and the average EC_50_ were 1.24 mM. The results indicated that OM 5930 may use as promising candidates in weed biological control for rice production.

## 1. Introduction

In Vietnam, rice (*Oryza sativa* L.) is one of the most important crops, accounting for a large proportion of the daily food intake of more than 90 million people. Rice is grown about 82% of cultivated land and plays an important role in the country’s economy. Vietnam has exported rice to about 150 countries and territories around the world, of which the Asian market accounts for 68.41%, followed by the African market (14.93%), the American market (6.54%), and the Oceania (5%) [1,2]. However, due to the crop intensification, an outbreak of pests on rice, including weeds has recently raised concerns. Weeds can reduce productivity by up to 60% due to competition for nutrients, light, and water without effective control measures [3]. In addition, weeds are also intermediary hosts for pathogens and insect pests on rice. Barnyardgrass (*Echinochola crus-galli* (L.) Beauv.) is the most competitive weed in rice production, resulting in a 70% yield loss from season-long interference [4], the competition of 25 barnyardgrass plants/m^2^ causes approximately 50% rice yield loss [5]. The sharp decline in rice yield caused by weeds, has led to the increased use of chemical herbicides by farmers due to their high efficiency, low cost, and time saving. However, overuse and overdose application of chemical herbicides through the years could be harmful to human health and the environment and may lead to herbicide resistance of weeds.

Currently, the provinces and cities in the Mekong Delta are promoting the implementation of the policy of restructuring the agricultural sector in the direction of improving added value and sustainable development, in which rice is one of the most important crops. Breeding and selection of new rice cultivars with high quality, resistance to pests and adverse conditions (low pH, saline, and drought), and capable of managing weeds by straw after harvesting has been interest of stakeholders, scientists, businesses, and farmers. Allelopathy is a biological phenomenon by which plants release chemicals that affect the germination and growth of other plants [6]. Plant-secreted substances are called allelochemicals. Studies conducted by [7,8] and [9] have shown that there are more than 90 allelochemicals identified in rice. These allelochemicals are found to be potent to pests, such as fungal diseases, insects, and, especially, inhibiting weed germination and development (Supplementary Material: Table S1).

The Cuu Long Delta Rice Research Institute (CLRRI) has so far bred 139 rice cultivars with names starting with OM. Our prior studies show OM rice cultivars exert inhibitory effect (EC_50_) on the development of shoots and roots of barnyardgrass at concentrations of 0.112 g/mL and 0.072 g/mL (OM 3536), and 0.091 g/mL and 0.062 g/mL (OM 5930), respectively [10]. Afterwards, there has been an allelochemical named *N-trans*-cinnamoyltyramine isolated from OM 5930 rice cultivar, capable of inhibiting barnyardgrass and red sprangletop at a concentration of 2.4 µM [11]. An efficient and low-cost total synthesis of this allelochemical was also successfully achieved by one step amidation from trans-cinnamic acid and tyramine [12]. However, the overview of allelopathy and allelochemicals in OM rice cultivars has not been fully discovered yet. This study aims to provide an insight into allelopathic properties of nine OM rice cultivars, including OM 5451, OM 380, OM 3536, OM 6976, OM 4498, OM 2395, OM N406, OM 5930, and OM 7347 and their associated allelochemicals as well as a combination of ultra-high-performance liquid chromatography mass spectrometry (UHPLC/MS) and XCMS software platform. This also contributes to the development and promotion of rice cultivars with allelopathic properties as well as reduction in herbicide-related issues in rice production. As herbicide-based weed management has become popular, our approach can be practiced for economically, environmentally friendly weed management in agricultural systems in not only Vietnam but also other rice growing areas in the world. 

## 2. Results and Discussion

### 2.1. Allelopathy of Rice on Barnyardgrass 

To establish an environmentally friendly and sustainable weed management strategy, allelopathy has been a topic of research for a long time. In addition, there have been a large number of rice cultivars found to inhibit several weed species when planted together in greenhouse conditions and laboratories [13]. There are approximately 191 of the 5000 rice cultivars capable of inhibiting the growth of weeds screened thus far. The United States Department of Agriculture’s Agricultural Research Service (USDA-ARS) screened more than 16,000 rice cultivars from 99 countries, of which 412 rice cultivars have allelopathic potential. In Egypt, there are more than 40 out of 1000 rice cultivars that showed inhibitory effects on the growth of barnyardgrass and flatsedge (*Cyperus difformis*) [13].

**Figure 1:**
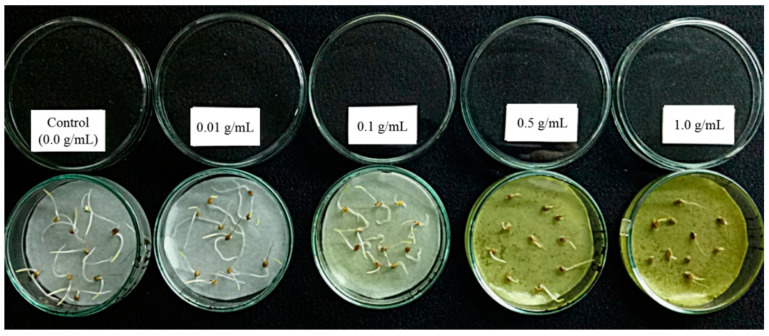
Effect of MeOH extracts (0.01, 0.1, 0.5 and 1.0 g·mL^−1^) from OM5930 rice cultivars on the shoot and root elongation of barnyardgrass (*Echinochloa crus-galli* (L.) Beauv) at 48 h after incubation.

Biological test results from the MeOH extracts of nine OM rice cultivars (OM 2395, OM 5451, OM 6976, OM 380, OM 5930, OM 4498, OM 3536, OM N406, and OM 7347) at concentrations of 0.01, 0.03, 0.1, 0.3, 0.5, and 1.0 (g mL^−1^) are presented in Table 1. From this result, the growth of barnyardgrass shoot under the effect of MeOH extracts from OM 5930, OM 5451, OM 6976, and OM 380 were immediately inhibitory at the lowest concentration of 0.01 g mL^−1^, inhibition increased when increasing concentration, rated from 5.60–98.77% corresponding with concentrations of 0.1 to 1.0 g mL^−1^. The OM 5930 extract caused the greatest inhibition comparing to the extracts of other rice cultivars (Figure 1). The extracts of OM 2395 and OM 3536 exhibited mild stimulation on barnyardgrass shoot growth (4.23–4.50%) at the concentration of 0.01 g mL^−1^. The effective dose requiring for more than 50.0% of barnyardgrass shoot inhibition was recorded at 0.3 g mL^−1^ for OM 5930 (57.39%) and OM 2395 (57.11%). However, at the highest concentration of 1.0 g mL^−1^, the extracts of OM 5930 and OM 4498 caused inhibition of more than 90% on the length of barnyardgrass shoot, while the extracts of remaining rice cultivars caused less inhibition (30.77–87.17%).

Table 2 shows that the MeOH extracts of six OM rice cultivars (OM 2395, OM 4498, OM 5930, OM 7347, OM 380, and OM N406) significantly inhibited the length of barnyardgrass roots (4.63–21.27%) at the lowest concentration of 0.01 g mL^−1^. Particularly, while OM 5451 extract showed no inhibition (9.58%), the extracts of OM 3536 and OM 6976 were stimulating the development of the barnyardgrass roots (10.44–19.62%) at this concentration. The OM 5930 extract inhibited nearly 50.0% barnyardgrass root (49.71%) at the concentration of 0.1 g mL^−1^ (Figure 1). At 0.3 g mL^−1^, OM 4498 and OM 5930 inhibited 74.58% and 66.93% barnyardgrass roots, respectively. The EC_50_ of nine OM rice cultivars on barnyardgrass root inhibition were 0.5 g mL^−1^ and above. At the highest concentration (1.0 g mL^−1^), the extracts of rice cultivars exhibiting more than 80% of barnyardgrass roots length inhibition were OM 6976 (86.56%), OM 4498 (92.83%), and OM 5930 (99.39%).

Many rice cultivars have been studied around the world for allelopathic characteristics and found to be able to inhibit the growth of some plant species when grown together [8,13,14]. The results of our study are also consistent with previous results about weed suppression activity of rice cultivars on barnyardgrass, in which, roots are always more sensitive than shoot to allelochemicals [15]. The average inhibition percentages of the nine OM rice cultivars on the growth of barnyardgrass shoot and root are 70.2% and 75.5%, respectively, examined allelopathy of 102 rice cultivars and found that the BR17 cultivar exhibited the greatest inhibitory activity on cress (*Lepidium sativum*), lettuce (*Lactuca sativa*), barnyardgrass, and jungle rice (*Echinochloa colona*) with the average inhibition on shoot and root are 39.5% [14]. Our results indicate that the nine OM rice cultivars may contain potential allelochemicals extracted by the MeOH solvent and affect the growth and development of barnyardgrass seedlings. 

### 2.2. Identification of Previously-Published Allelochemicals

**Figure 2:**
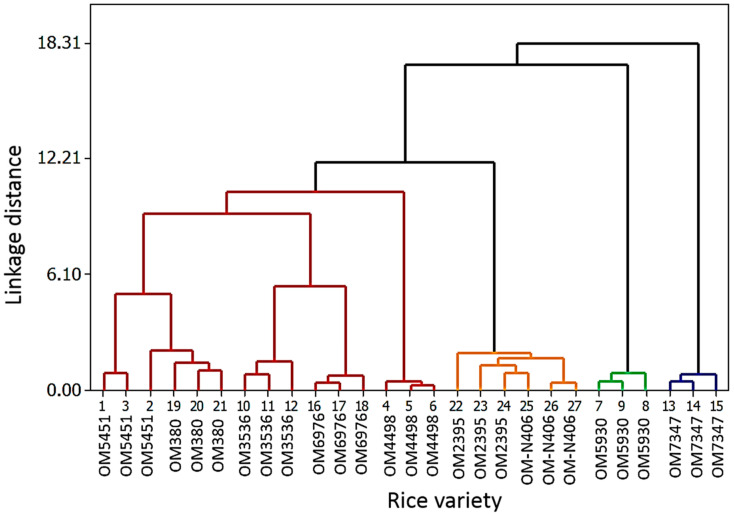
Dendrogram for the rice cultivars ((OM2395, OM5451, OM6976, OM380, OM5930, OM4498, OM3536, OM-N406, and OM 7347) from the hierarchical cluster analysis.

The above nine allelopathic rice cultivars were used for initial screening to identify allelochemicals capable of inhibiting the growth and development of barnyardgrass. An untargeted metabolomics approach was employed to identify allelochemicals in MeOHic extracts of these rice cultivars. We focused on identification of the class of compounds which have previously been reported to exert allelopathic activities (Table S1) using UHPLC-MS combined with XCMS Online platform. The two*-*dimensional hierarchical cluster analysis (HCA 2D) diagram was used to identify relative similarity in allelochemical profiles among the rice samples, and the results were graphically presented as a dendrogram (Figure 2).

Figure 2 displays the dendrogram which shows four clusters of rice samples grouped by similarities in the studied allelochemical contents using Ward’s method [16]. The HCA 2D diagram is used to compare the similarities and differences between the distances of two points in the Euclidean distance of secondary metabolites detected during the metabolism of nine OM cultivars (OM 2395, OM 5451, OM 6976, OM 380, OM 5930, OM 4498, OM 3536, OM N406, and OM 7347). The Euclidean distance is directly proportional to the difference between biochemical components in those rice cultivars. The HCA analysis is based on the Euclidean distance with each cluster divided based on similarities and differences with each functional group of biochemical components. Based on Figure 2, OM 5451, OM 380, OM 3536, OM 6976, and OM 4498 (red pedigree tree branches) have much more biochemical components than OM 2395 rice cultivars, OM N406, OM 5930, and OM 7347 (genealogy branches orange, green, and blue).

**Figure 3:**
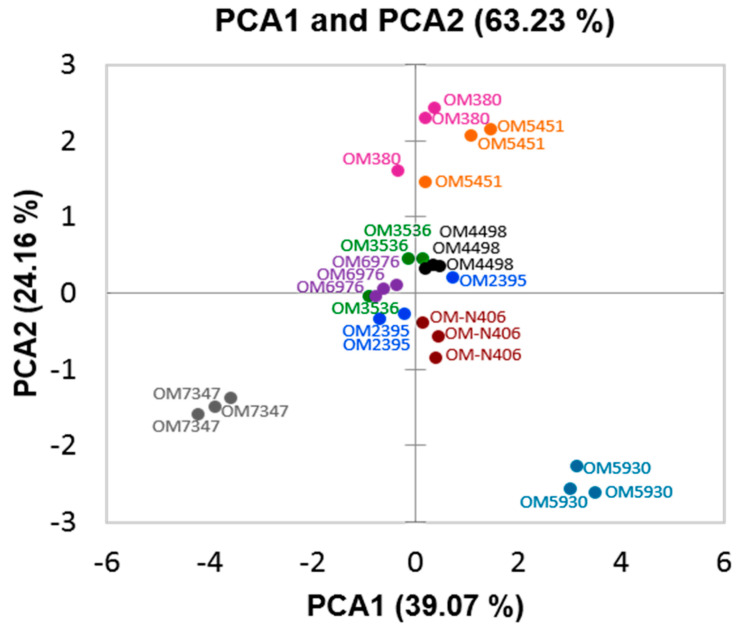
Principal component analysis of allelochemical data of the rice cultivars (OM2395, OM5451, OM6976, OM380, OM5930, OM4498, OM3536, OM-N406, and OM 7347).

The principal component analysis was used to evaluate the allelochemical data in the rice cultivars determined by UHPLC-MS. As shown in Figure 3, principal component one explains up to 39.07% of the total variance, and represents cinnamic acid, coumarin, and 7-oxostigmasterol. The OM 5930 cultivar, which contained significantly higher levels of these compounds, clusters separately from the others. Principal component two explaining 24.16% represents salicylic acid. The PCA graph showed 63.23% of the total variance in the allelochemical data set (Figure 3). 

**Figure 4:**
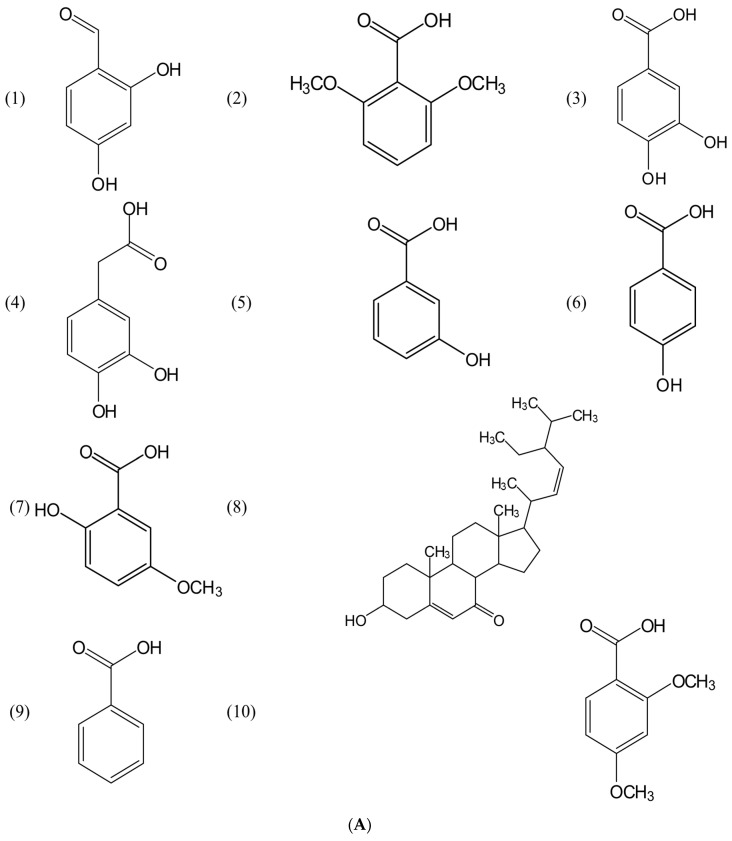

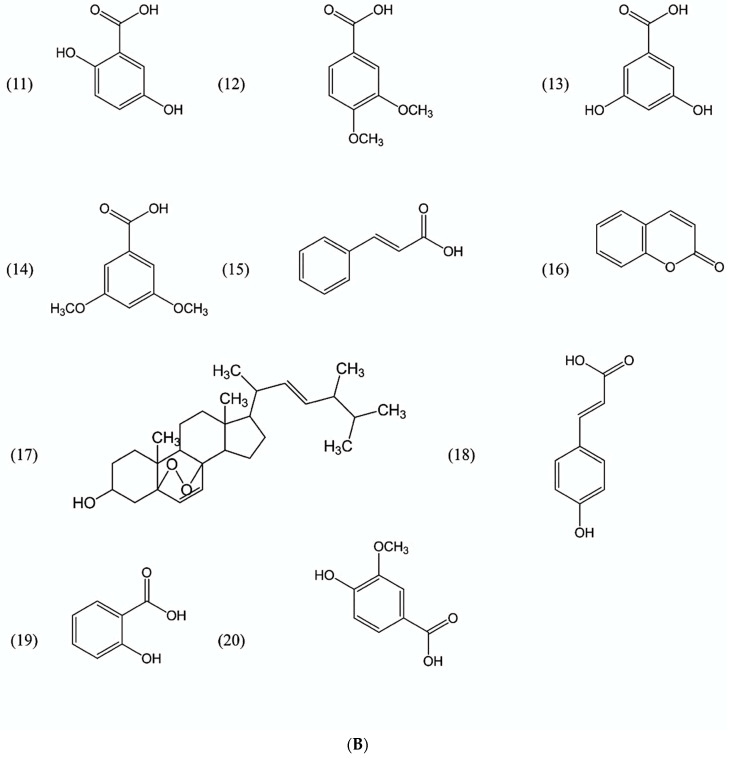
(**A**) Chemical composition of 10 allelochemicals in 9 OM rice cultivars identified as 2,4-dihydroxybenzaldehyde (1); 2,6-dimethoxybenzoic acid (2); 3,4-dihydroxybenzoic acid (3); 3,4-dihydroxyphenylacetic acid (4); 3-hydroxybenzoic acid (5); 4-hydroxybenzoic acid (6); 5-methoxysalicylic acid (7); 7-oxostigmasterol (8); benzoic acid (9); and 2,4-dimethoxybenzoic acid (10). (**B**) Chemical structure of 10 allelochemicals in 9 OM rice cultivars identified as 2,5-dihydroxybenzoic acid (11); 3,4-dimethoxybenzoic acid (12); 3,5-dihydroxybenzoic acid (13); 3,5-dimethoxybenzoic acid (14); cinnamic acid (15); coumarin (16); ergosterol peroxide (17); *p*-hydroxycinnamic acid (18); salicylic acid (19); and vanillic acid (20).

Through the UHPLC-MS analysis, over 31,000 substances have been analyzed in nine rice cultivars. However, only about 750 substances were identified to have allelopathic activity based on the result obtained by the XCMS software (Table S2). After screening the presence of allelochemicals that have plant growth activity from the biochemical analysis of nine rice cultivars with those 751 substances, and their role in inhibiting plant growth and development available in databases and research articles, there are 20 allelochemicals presented in nine rice cultivars (Table 3; Figure 4A,B), including 2,4-dihydroxybenzaldehyde (1), 2,6-dimethoxybenzoic acid (2), 3,4-dihydroxybenzoic acid (3), 3,4-dihydroxyphenylacetic acid (4), 3-hydroxybenzoic acid (5), 4-hydroxybenzoic acid (6), 5-methoxysalicylic acid (7), 7-oxostigmasterol (8), benzoic acid (9), 2,4-dimethoxybenzoic acid (10), 2,5-dihydroxybenzoic acid (11), 3,4-dimethoxybenzoic acid (12), 3,5-dihydroxybenzoic acid (13), 3,5-dimethoxybenzoic acid (14), cinnamic acid (15), coumarin (16), ergosterol peroxide (17), *p-*hydroxycinnamic acid (18), salicylic acid (19), and vanillic acid (20). Four levels of metabolite identifications were previously summarized by [17]. In the present study, the allelochemicals were putatively identified based on the similarities of their mass spectra with those in a public spectral library (METLIN), denoting that the identification level is two.

The number of allelochemicals present in each rice cultivar is one of many factors affecting its weed inhibitory activity. In this study, the number of allelochemicals in OM 4498, OM–N406, and OM 5930 are 14 (70%), 13 (65%), and 13 (65%), respectively. In particular, there are nine similar allelochemicals presented in those three rice cultivars as 2,5-dihydroxybenzoic acid, 3,4-dihydroxybenzoic acid, 3-hydroxybenzoic acid, 4-hydroxybenzoic acid, 7-oxostigmasterol, 3,5-dihydroxybenzoic acid, cinnamic acid, *p-*hydroxycinnamic acid, and salicylic acid. Similarly, the number of allelochemicals in OM 5451, OM 6976, and OM 2395 are 9 (45%), in OM 3536 is 10 (50%), and in OM 380 is 7 (35%). The OM 7347 cultivar contains only two allelochemicals (10%). 

Additionally, the presence of each allelochemical in the nine rice cultivars was also explored. There were 13 different phenolic acid compounds from rice husks such as benzoic acid, 4-hydroxybenzoic acid, protocatechuic acid, gallic acid, vanillic acid, syringic acid, salicylic acid, 2,5-dihydroxybenzoic acid, *β*-resorcylic acid, *p-*coumaric acid, caffeic acid, ferulic acid, and 3,5-dimethoxy-4-hydroxycinnamic acid analyzed through gas chromatographic system [18]. Of these, *p-*coumaric acid was the most commonly found compound, while the least ones were ferulic acid and vanillic acid. Similar to the results on nine OM rice cultivars, *p-*hydroxycinnamic acid was found in all the samples examined, following by cinnamic acid (88.9%), 7-oxostigmasterol (77.8%), 3-hydroxybenzoic acid (66.7%), 4-hydroxybenzoic acid (66.7%), salicylic acid (66.7%), 3,4-dimethxybenzoic acid (66.7%), 3,4-dihydroxybenzoic acid (55.6%), 2,4-dimethoxybenzoic acid (66.7%), 2,5-dihydroxybenzoic acid (55.6%), 3,5-dihydroxybenzoic acid (55.6%), 3,4-dimethoxybenzoic acid (55.6%), and 3,5-dimethoxybenzoic acid (55.6%). The remaining allelochemicals with detection frequency ranging from 11.1% to 33.3% include 3,4-dihydroxyphenylacetic acid (33.3%), vanillic acid (33.3%), 2,6-dimethoxybenzoic acid (22.2%), 2,4-dihydroxybenzaldehyde (11.1%), 5-methoxysalicylic acid, benzoic acid (11.1%), coumarin (11.1%), and ergosterol peroxide (11.1%).

As demonstrated in Table S1, 3-hydroxybenzoic acid, 4-hydroxybenzoic acid [13], 7-oxostigmasterol, ergosterol peroxide [19], benzoic acid [18], salicylic acid [18,20,21], and vanillic acid [13,21,22] have previously been identified as allelochemicals exhibiting inhibitory effects against weeds.

Dated back in 2006, Macias and colleagues [19] successfully identified and isolated bioactive steroids from parts of rice. Most of these phytotoxic compounds, including ergosterol peroxide and 7-oxostigmasterol, reportedly exerted strong inhibitory effects on barnyardgrass. This is also the first report on allelopathic activity of steroids on weed. According to Li et al. (2010), pea (*Pisum sativum*) was completely inhibited when using gallic acid at a concentration of 1 mM, hydroxybenzoic acid at a concentration of 2 mM and the rest were 2.5-dihydroxybenzoic acid and gallic acid with reduced inhibitory effect at both 1 mM and 2 mM [23]. Chou et al. (1991) reported that 4-hydroxybenzoic acid and salicylic acid completely inhibited the germination of lettuce seeds and alfalfa (*Medicago sativa*) at concentrations of 0.5 and 1.5 mM, respectively [24]. The previous results also revealed that vanillic acid at 100 and 200 ppm almost inhibited lettuce (97.3%), radish (*Raphanus sativus*) roots (33.0–62.6%) and barnyardgrass (36.1–80.6%), and indicated an evidence that the three compounds as 4-hydroxybenzoic acid, salicylic acid, and vanillic acid have great potentials to inhibit 94%, 50%, and 73% of barnyardgrass germination at concentration of 1mM, respectively [25].

However, of those 20 identified allelochemicals, 12 were previously identified in rice as 2,5-dihydroxybenzoic acid, benzoic acid [18], 3,4-dihydroxybenzoic acid [18,24], 3-hydroxybenzoic acid, 7-oxostigmasterol, ergosterol peroxide [19], 4-hydroxybenzoic acid [26], cinnamic acid [27], 3,4-dihydroxyphenylacetic acid [6,13], *p-*hydroxycinnamic acid [6,21], salicylic acid [18,20,21], and vanillic acid [13,21,22].

In addition, not only in rice, the above compounds were also present in some other crops, for example, benzoic acid, 3-hydroxybenzoic acid, vanillic acid, and *p-*coumaric acid in corn (*Zea mays*), and ferulic acid, vanillic acid, phenylacetic acid, *p-*coumaric acid, *p-*hydroxybenzoic acid, and salicylic acid in rye (*Secale cereale*) [28]. In particular, salicylic acid and benzoic acid inhibit the growth of lettuce at 25–50 ppm. Fernandez et al. (2006) identified 4-hydroxybenzoic acid, vanillic acid, cinnamic acid, *p-*coumaric acid, and benzoic acid in pine (*Pinus halepensi*) [29]. Extracts of leaves, flowers, and stems of the phoenix (*Delonix regia*) contain 3*-*hydroxybenzoic acid, vanillic acid, 2,5-dihydroxybenzoic acid, and *p*-coumaric acid exhibited an inhibitory effect on over 30% on the germination of lettuce seeds at the lowest concentration of 10 ppm compared to the control [23].

The remaining eight allelochemicals (3,4-dimethoxybenzoic acid, 3,5-dimethoxybenzoic acid, 3,5-dihydroxybenzoic acid, 2,4-dihydroxybenzaldehyde, 2,6-dimethoxybenzoic acid, 5-methoxysalicylic acid, 2,4-dimethoxybenzoic acid, and coumarin) have not been previously found in rice but are present in nine OM cultivars. In particular, 2,4-dimethoxybenzoic acid is one of the chemical components found in pine [29] and in extracts of crabgrass (*Digitaria sanguinalis*) roots and crabgrass exudates. This compound adversely affected the growth of other plants at the concentrations of 0.16 to 8.10 µg g^−1^ [30]. Coumarin was found to inhibit the germination of alfalfa, Italian ryegrass (*Lolium multiforum*), and velvetweed (*Abutilon theophrasti*) [31] and was identified as an allelochemical in sweet vernal grass *(Anthoxanthum odoratum*) [32]. Plants with high coumarin content have been used in intercropping systems to inhibit germination and growth of black-jack (*Bidens pilosa*) [7,33,34]. Authentic coumarin had an EC_50_ of 23.3 µmol L^−1^ and the crude extract posing 50% inhibition on radicle growth of lettuce seedlings contained coumarin at a concentration of 9.7 µmol L^−1^ [35]. A study by Choi et al. (2016) revealed 92 benzene derivatives capable of inhibiting the extension of cabbage (*Brassica campestris*) roots [36]. There are 14 benzene derivatives such as 2,4-dihydroxybenzaldehyde, 2,6-dimethoxybenzoic acid, 3,4-dihydroxybenzoic acid, 4-hydroxybenzoic acid, 5-methoxysalicylic acid, benzoic acid, 2,4-dimethoxybenzoic acid, 2,5-dihydroxybenzoic acid, 3,4-dimethoxybenzoic acid, 3,5-dihydroxybenzoic acid, 3,5-dimethoxybenzoic acid, and salicylic acid inhibited over 70% of the germination ability of canola (*Brassica campestris*) seeds at concentrations below 100 g mL^−1^.

In summary, there were total of 20 plant growth inhibitors identified in 9 OM rice cultivars, 12 of them already found in rice previously and other 8 were either identified in other crops or tested to have allelopathic activities. The 4, rice cultivars as OM 5930, OM 4498, OM 3536, and OM N406 accounted for 50% or more of the number of allelochemicals as compared to the total number of identified allelochemicals, followed by the cultivars as OM 2395, OM 5451, OM 6976, and OM 380 accounting for more than 35% to 45%. The OM 7347 cultivar had the lowest number of identified allelochemicals (10%).

In order to create a foundation for the next research directions, it is necessary to have information about the presence of predominant allelochemicals in each rice cultivar in order to select rice cultivars with high allelopathic potential for further investigation and identification, additional allelochemicals capable of inhibiting weed towards sustainable and environmentally friendly management (Table 4 and Table 5). Eight allelochemicals, both inhibitory and stimulating, were putatively determined (based on their *m*/*z*) by UHPLC-MS in the rice extracts in positive and negative ion modes. Their molecular formulae, mass errors, and retention times are demonstrated in Table 4. A compound with [M + H]^+^ ion at *m*/*z* 159.0512 was assigned as allantoin. This allelochemical was found in all the nine rice cultivars, except OM 5930 and OM 6976 (Table 5). Our study also reported the identification of four phenolic allelochemicals, namely benzoic acid, cinnamic acid, vanillic acid, and salicylic acid, which were detected with [M + H]^+^ ions at *m*/*z* 123.0441, 149.0596, 169.0496, and 137.0244, respectively. The list also includes a phenylpropanoid, namely coumarin with [M + H]^+^ ion at *m*/*z* 147.0440. Two compounds with [M + H−H_2_O]^+^ ions at *m*/*z* 409.3471 and 411.3269, were assigned as 7-oxostigmasterol and ergosterol peroxide. 

The results in Table 5 showed that the rice cultivars significantly differed on the relative abundances of eight predominant allelochemicals at *p* < 0.05. Among these compounds, coumarin, 7-oxostigmasterol, and salicylic acid were identified in all rice cultivars. The study also yields evidence that ergosterol peroxide, the most important allelochemical in term of weed suppression [19], is detected in only OM 5930. The cultivars OM 5451, OM 380, OM 6976, OM N406, and OM 5930 contains significantly higher levels of cinnamic acid compared to OM 3536 and OM 2395. The results also revealed that OM 5451 and OM 5930 contain almost triple as much amount of salicylic acid as OM 380. Furthermore, in those eight predominant allelochemicals, allantoin was reported to significantly stimulate the germination and development of barnyardgrass at concentrations of 30–500 µg g^−1^ soil [37]. Specifically, allantoin not only plays a role in metabolism and storage of nitrogen in rice, but also an intermediary role for the interaction between rice and microorganisms in soil, and its concentrations above 500 µg g^−1^ do not increase soil microbial population. The levels of allantoin in OM 5451 and OM 380 were found to be significantly higher than those of the other cultivars but not in OM 5930. 

**Figure 5:**
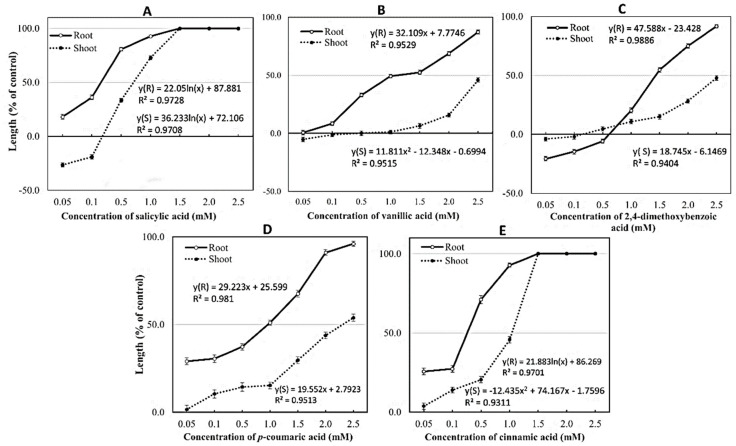
Effect of five allelochemicals confirmed in OM 5930 rice cultivar on the shoot and root growth of mustard green at concentrations of 0.05, 0.1, 0.5, 1.0, 1.5, 2.0, and 2.5 mM. (**A**) salicylic acid, (**B**) vanillic acid, (**C**) 2,4-dimethoxybenzoic acid, (**D**) *p*-coumaric acid, and (**E**) cinnamic acid.

Coming back to Table 1 and Table 2, the OM 4498 has the second largest inhibition rate at the concentration of 1g mL^−1^. However, it is in the center of the PCA scores plot and close to other seven cultivars, but not close to OM 5930 (Figure 3). This may be understood as the inhibition rate of OM rice cultivar on barnyardgrass is not only depended on the abundance of allelochemicals contained in the rice but also depended on which allelochemical and how it is important to make the rice more allelopathic. As seen in Table 5, the three important allelochemicals, cinnamic acid, coumarin, and 7-oxostigmasterol occur in the OM 4498 at the significant different lower levels in comparison with the OM 5930; benzoic acid and egosterol peroxide occur in the OM 5930 but not in OM 4498. However, salicylic acid is the allelochemical that causes greatest allelopathic plant growth inhibition (Figure 5) accounting for the highest level in OM 4498 in comparison to other rice cultivars including OM 5930. Those explains why even the PCA of OM 4498 is quite far from the OM 5930 but its allelopathic potential is closer to the OM 5930. Based on the understanding of allelopathic activities and the levels of the above eight predominant allelochemicals in the nine rice cultivars, it is presumable that the cultivar of OM 5930 may possess the highest weed inhibitory effect. This is also in agreement with the results in Section 2.1 (allelopathy of rice on barnyardgrass) that OM 5930 inhibited 98.77% on shoot and 99.39% on root of barnyardgrass, significantly statistically different at *p* < 0.01 as compared with other rice cultivars.

### 2.3. Confirmation of Allelochemicals in OM 5930 Rice Cultivar

The most allelopathic OM 5930 rice cultivar was chosen after having the data from the Section 2.1 and Section 2.2 above for further confirming of feature allelochemicals such as salicylic acid, vanillic acid, *p*-coumaric acid, 2,4-dimethoxybenzoic acid, and cinnamic acid. Quantitative measurement by comparing the retention time of these allelochemicals in OM 5930 rice sample with the corresponding allelochemical standards are shown in Table 6. The identification of the five allelochemicals were based on the use of authentic analytical standards, indicating that the identification level is one. The results revealed that OM 5930 rice cultivar contains five allelochemicals with corresponding ingredients in 100 g of fresh rice as salicylic acid (5.0076 mg), vanillic acid (0.1246 mg), *p*-coumaric acid (0.1590 mg), 2,4-dimethoxybenzoic acid (0.1045 mg), and cinnamic acid (3.3230 mg). 

**Figure 6:**
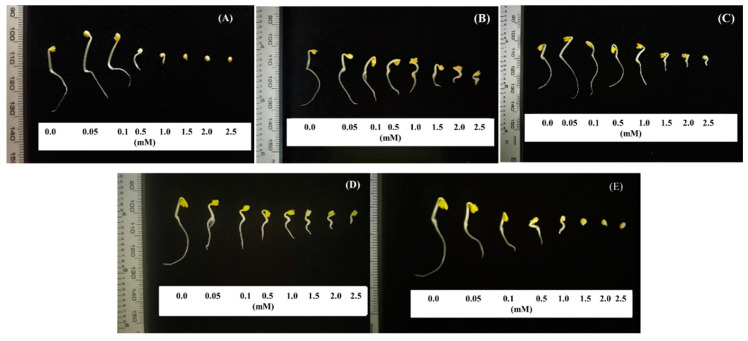
Effects of (**A**) salicylic acid, (**B**) vanillic acid, (**C**) 2,4-dimethoxybenzoic acid, (**D**) *p*-coumaric acid, and (**E**) cinnamic acid on shoot and root growth of mustard green seedlings following 48 h of incubation at various concentrations.

Those five allelochemicals at different concentrations showed either inhibitory or stimulating effects on the growth and development of the shoot and root length of mustard green. *p*-coumaric acid and cinnamic acid inhibited the growth of mustard green root and stem from concentrations of 0.05 and 0.1 mM, respectively (Figure 5A, Figure 6A, Figure 5E and Figure 6E); lower inhibitory exhibitions were salicylic acid (0.05 and 0.5 mM), vanillic acid (0.1 and 1.5 mM), and 2,4-dimethoxybenzoic acid (1.0 and 0.5 mM) (Figure 5B–D). The inhibition increases with increasing concentration (Figure 5 and Figure 6). Some other research results also noted that vanillic acid at 4 ppm inhibited 11.4% tomato (*Solanum lycopersicum*) germination [38]. Choi et al. (2016) also reported that 2,4-dimethoxybenzoic acid at a concentration of 500 mg L^−1^ completely inhibited the growth of mustard greens [36]. Cinnamic acid is effective even at concentrations below 0.01 mM, indicating the ability of cucumber toxicity in field conditions [39]. *p*-coumaric acid has been previously identified to be presented in rice plants and determined to express weed resistance to barnyardgrass [6,21].

At 1.5 mM, salicylic acid and cinnamic acid inhibited 100% of the growth of both roots and mustard greens. While at the highest concentration of 2.5 mM, the remaining substances only inhibited an average of 49.2–91.7% of mustard green seedlings, namely vanillic acid (45.9% and 87.3%), 2,4-dimethoxybenzoic acid (47.8% and 91.7%), and *p*-coumaric acid (53.9% and 96.1%). Similarly, Einhellig and Rasmussen (1979) pointed out that vanillic acid at a concentration of 2.5 mM inhibited the seedling growth by 71% compared to the control after 24 h of follow-up [40]. Through linear and polynomial regression analysis, the EC_50_ of five identified allelochemicals in OM 5930 on the root and shoot growth of mustard were salicylic acid (0.24 and 1.26 mM), vanillic acid (1.32 and 0.51 mM), 2,4-dimethoxybenzoic acid (1.54 and 3.00 mM), *p*-coumaric acid (0.83 and 2.41 mM), and cinnamic acid (0.31 and 1.05 mM).

Our results were particularly noted that salicylic acid at concentrations of 0.05 and 0.1 mM stimulated the development of mustard greens by 26.5% and 19.1%, respectively. 2,4-Dimethoxybenzoic acid also stimulated 4.0% and 1.9% the shoot growth of mustard greens at concentrations of 0.05 and 0.1 mM; 20.7%, 14.1%, and 5.9% the root growth of mustard greens at concentrations of 0.05, 0.1, and 0.5 mM, respectively. This is also consistent with the study of [41] that the effects of phenols such as ferulic acid, gallic acid, *p*-coumaric acid, *p*-hydroxybenzoic acid, and vanillic acid on the germination and growth of various weeds at different concentrations were different, they can stimulate the growth of weeds at concentrations less than 1 mM. In addition, Choi et al. (2016) reported that salicylic acid at concentration above 1 mM inhibits germination and 10–100 µM inhibits the growth of *Arabidopsis thaliana* [36].

Plant roots are susceptible to allelochemical stress during early seedling growth [42,43] due to high metabolic activity and the start of lignification at this stage [44]. Studies with phenolic acids including salicylic acid, vanillic acid, 2,4-dimethoxybenzoic acid, *p*-coumaric acid, and cinnamic acid have shown inhibitory effects on root growth greater than in shoot growth in different plants species, [39,45,46,47,48,49] including mustard green [36]. This study further confirmed the susceptibility of mustard green for those five compounds, which significantly reduced 50% the shoot and root growth of mustard green at the average concentrations of 1.64 and 0.85 mM, respectively. 

## 3. Materials and Methods

### 3.1. Extraction and Allelopathy Bioassays

#### 3.1.1. Plant Material

The nine OM rice cultivars (OM 2395, OM 3536, OM 4498, OM 5451, OM 5930, OM 6976, OM 7347, OM 380, and OM N406) were collected from the net house at CLRRI in Can Tho, Vietnam after 60 days of sowing. Barnyardgrass seeds were collected from experimental fields at CLRRI. 

#### 3.1.2. Extraction of Nine OM Rice Cultivars by Methanol (MeOH) Separation Method

The stems, leaves, and roots of each OM rice cultivar after being harvested from the net house were carefully prepared to completely remove soil in rice roots and dirt. Clean scissors were used to shred 100 g of fresh stem, leaf, and root tissues of rice together into a triangle flask with one liter of MeOH:H_2_O (3:2, *v*/*v*) corresponding to 600 mL MeOH mixed into 400 mL of distilled water for 48 h. The flask was agitated twice daily during immersion to mix thoroughly and help the rice materials completely submerge in the solvent mixture. The first extract was collected by filtration, using a 320 mL Buchner porcelain funnel (90 mm diameter, Fisher Scientific, Waltham, MA, USA). The sample was stored in the refrigerator (−4 °C). The residue was then extracted with 700 mL of MeOH for 48 h and the combined extract (1.7 L) was evaporated at 42 °C using a vacuum rotary evaporator to obtain 400 mL of the final aqueous extract. The extract was then titrated with 1 mL of phosphate buffer to achieve pH = 7.0. An amount of 40 mL of this extract (corresponding to 10 g of fresh rice tissue) was used to investigate allelopathic properties [11].

#### 3.1.3. Bioassays 

The allelopathic method was done equally for nine OM rice cultivars. Each experiment was conducted in six different concentrations (0.01, 0.03, 0.1, 0.3, 0.5, and 1.0 g mL^−1^), arranged 10 seeds /treatment, and each treatment was performed in triplicate. The seeds used in the assay were soaked for 48 h and incubated for another 36 h to collect newly germinated seeds. A micropipette was used to transfer the extract at different concentrations into corresponding Petri dishes (50 mm diameter) lined up with filter paper under laboratory conditions. Each of these Petri dishes was placed in a fume hood at room temperature (25 °C) until the solvent in the extract was completely evaporated. The filter paper in each Petri dish was further moistened with 1.0 mL of 0.05% Tween 20, using only 0.05% Tween 20 for the control experiments. Next, 10 newly germinated seeds of barnyardgrass were added into Petri dishes and the dishes were carefully wrapped. The experiment was conducted in a stable temperature at 25 °C in the dark. After 48 h of incubation, shoot and root lengths were measured using an electronic ruler [11].

#### 3.1.4. Bioassay Data Analysis

The inhibition percentage (%) was calculated as follows:

*I* = [(*L*1 − *L*2*)/L*1] × 100

in which, *I* stand for the percentage of inhibition; *L*1 is the average length of roots or shoots of the control plant; and *L*2 is the average length of roots or shoots of treated plants.

The data were processed by Microsoft Office Excel 2013. SPSS (version 20.0) was used to analyze ANOVA one factor, using letter symbols to compare the differences between the average results of all treatments by the Duncan Multiple Range Test (Duncan test) [11].

### 3.2. Identification of Previously Published Allelochemicals Using Cloud-Based Metabolomics Platform

#### 3.2.1. Rice Materials

In a previous unpublished study, we already found that the OM 5930 rice cultivar releases a certain amount of N-trans-cynamoylthyramine, an allelochemical identified by [11], at the growth development stage of 10–20 days really equal to 55–65 days. Other scientists have also reported that the highest concentration and diversity of secondary metabolites were found during the first 2 weeks after rice seeding [50]. Several classes of carbohydrates, amino acids, phenolic acids, cytokinins, alkyl resorcinols, momilactone B, and flavones have been determined from rice root exudates as allelochemicals [50,51,52]. It has been shown that a total of 7.6 n moles of 3-Isopropyl-5-cetoxycyclohexene-2-one-1, momilactone B, and 5,7,4’-trihydroxy-3’,5’-dimethoxyflavone were exuded from living roots of each rice seedling into the environment at 10 days after seedlings were transplanted [53]. Therefore, rice seedlings at 10 days after sowing may produce enough allelochemical amount for chromatographic detection.

In this study, seeds of OM rice cultivars (2395, 3536, 4498, 5451, 5930, 6976, 7347, and N406) were soaked, germinated, and then placed in 10 cm diameter Petri dishes (100 seeds/dish). The Petri dishes were placed in the plant growth chamber at 25 °C, light intensity 80–100 μE m^−2^ s^−1^, 12 h of photosynthesis. After 10 days, rice stems, leaves, and roots were collected, rinsed with distilled water, and drained by absorbing with filter paper. About 10 g for each rice cultivar were placed in a liquid nitrogen solution, then ground into powder. The rice powder obtained was stored in 15 mL plastic centrifuge tubes, carefully noted, wrapped in aluminum foil and kept in refrigerator at 4 °C for the UHPLC-MS analysis.

#### 3.2.2. UHPLC/MS Analytical Method

One g of each rice powder was mixed with 40 mL of MeOH in a 50 mL screw-capped test tube. The tube was vortexed thoroughly for 1 min, followed by sonication for 90 min. The mixture was then kept in the freezer (−20 °C) overnight. The sample was removed from the freezer and kept at room temperature for about 60 min, vortexed for 1 min then centrifuged for 6 min at 13,000 rpm (14,700× *g*). The supernatant was collected and filtered through 0.2 μm Whatman Anotop filter (GE Healthcare, Darmstadt, Germany) and transferred to an LC vial prior to injection into UHPLC/MS.

Multivariate data analysis was used to compare the differences between biochemical components of nine rice cultivars identified through the UHPLC/MS. The method of principal component analysis (PCA) is used to assess the data of allelochemicals in the rice cultivars.

The UHPLC/MS analysis was performed on a Bruker maXis impact quadrupole-time-of-flight mass spectrometer coupled with a Waters ACQUITY UPLC system. Separation was obtained on a Waters C18 column (2.1 by 100 mm, BEH C18 column with 1.7 µm particles) using a linear gradient of 95%:5% to 30%:70% eluent A:B (A: 0.1% formic acid, B: acetonitrile) over 30 min. Between 30–33 min, the linear gradient was increased from 70% to 95% B, and maintained at 95% B for 3 min. The percentage of B was also maintained at 5% from 36–40 min. The flow rate was 0.56 mL min^−1^ and the column temperature was 60 °C. Mass spectrometry was performed in the negative (or positive) electrospray ionization mode with the nebulization gas pressure at 43.5 psi, dry gas of 12 L min^−1^, dry temperature of 250 °C and a capillary voltage of 4000 V. Mass spectral data were collected from m/z of 100 and 1000, and were auto*-*calibrated using sodium format after data acquisition. 

#### 3.2.3. Data Analysis Platform

The mass spectrometric data obtained from UHPLC-MS were processed using XCMS online in pair-wise job mode. The CDF files generated from UHPLC-MS were uploaded into XCMS Online (https://xcmsonline.scripps.edu/). The automated data process includes peak detection, peak grouping, spectra extraction, and retention time alignment. Pair comparisons were used for two groups (i.e., rice extracts and MeOH control blanks). Features were listed in a feature list table, containing their extracted ion chromatographic peak areas. The default XCMS parameters set for UHPLC-MS were employed with tolerance for database search set to 5 ppm. Putative identification of rice allelochemicals was performed by the integration of the METLIN database into XCMS Online. Unpaired parametric t*-*test (Welch *t-*test) with *p-*value ≤ 0.005 and fold change ≥ 2 were applied to determine features with filtered intensity ≥ 10,000. The allelochemical identification was based on the results of the up-regulated features. The Bio-Source was set as “rice”, and other parameters for XCMS processing of our data acquired by UHPLC-MS were set as default. Only features with filtered intensity ≥ 10,000 in the extract while ≤ 5000 in the MeOH control blank were reported. These parameters helped remove isotopes and noise, prioritizing the aligned features whose levels vary significantly between the rice extracts and MeOH blank samples. 

#### 3.2.4. Statistical Analysis

One-way analysis of variance (ANOVA) was carried out to determine difference of the allelochemical relative abundance (peak area) between the rice cultivars. Comparisons of means were made using Minitab program (Minitab 16, Minitab Inc., State College, PA, USA). Multiple comparisons were determined using the Tukey’s Studentized Range HSD test at a significance level of *p* < 0.05. Principal component analysis and hierarchical cluster analysis were performed using Minitab.

### 3.3. Confirmation of Allelochemicals in OM 5930 Rice Cultivar

#### 3.3.1. Sample Preparation

Salicylic, cinnamic, 2,4-dimethoxybenzoic, *p*-coumaric, and vanillic acids were provided by Shanghai Energy Chemicals (Shanghai, China). Their purity is above 98% determined based on HPLC analysis technique. Acetonitrile (ACN) and MeOH solvents for HPLC standard were purchased from Merck K GaA (Darmstadt, Germany). The pure water was filtered through the US Milli-Q filtration system (Millipore, Bedford, MA, USA). 

360 mL aqueous extract of the most OM rice cultivar collecting after the section of “Extraction and Allelopathy Bioassays**”** was then partitioned by liquid /liquid extraction with the volumn of EtOAC in the rice extract (2:1, *v*/*v*) to collect ethyl acetate and aqueous fractions. The EtOAC fraction was then purified by chromatographic columns of Silica (60 g, silica gel 60, 70–230 mesh; Merck) eluting stepwise with n-hexane containing increasing amounts of ethyl acetate (10% per step, *v*/*v*; 100 mL per step), Sephadex^TM^ LH-20 column (50 g, Amersham Pharmacia Biotech, Buckinghamshire, UK) eluting with 20%, 40%, 60%, and 80% (*v*/*v*) aqueous MeOH (50 mL per step) and 100% MeOH (100 mL), and C18 Sep-Pak cartridges (Waters, Milford, MA, USA) eluting with 20%, 40%, 60%, and 80% (*v*/*v*) aqueous MeOH and 100% MeOH (15 mL per step). The final phase in the form of 20% MeOH solution collected from C18 SPE purification was diluted in a certain proportion depending on the concentration of the sample solution and in accordance with the running conditions of the HPLC system. All standard and analytical sample solutions were filtered through a 0.21 µm membrane Anotop filter prior to analysis.

Each 20 mg of standard sample was dissolved in 1.0 mL of MeOH solvent to obtain a 20,000 ppm stock standard solution mixture. The calibration curve was constructed after dilution of the standard solution with 100% MeOH solvent. Reference solutions of standards at different concentrations depend on the specific standard, analyzed by the HPLC/UV-Vis system. The regression equation was calculated based on the formula y = ax + b, where y and x correspond to the ratio of peak (area of substance peak) and substance concentration.

#### 3.3.2. Chromatographic Conditions of HPLC/UV-Vis and Its Optimization

The HPLC system of Agilent 1260 series with G1311C quadrupole pump, G2260A autosampler pump, G1316A column thermostat, DAD G1315D probe was used to analyze the samples. XDB-C18 column (150 mm by 4.6 mm, 5 µm; Agilent Technologies, Santa Clara, CA, USA) equipped with column guard (3.9 mm by 20 mm, C18, 5 µm). The eluting solvent systems consisted of MeOH (A) and H_2_O + 0.1% formic acid (B) were 30:30 (*v*/*v*) for 2,4-dimethoxybenzoic acid, *p*-coumaric acid, and vanillic acid; 50:50 for salicylic acid and consisted of Acetinitrile (A) and H_2_O + 0.1% formic acid (B) was 20:80 (*v*/*v*) for cinnamic acid. The analytical system eluted with a flow rate of 0.5 mL min^−1^ and a range of UV waves scanned from 200 to 400 nm. The chromatographic process was run on specialized software by Agilent. All allelochemical standards were completely separated in a 30-minute period. The peaks in the chromatography spectrum of the rice sample solutions were determined by comparing their retention times with the corresponding allelochemical standards. 

#### 3.3.3. Molecular Bioassay

Each identified growth inhibitory compound was dissolved in 1.0 mL of MeOH, and compound concentrations of 0.05, 0.1, 0.5, 1.0, 1.5, 2.0, and 2.5 mM were added onto a sheet of filter paper (No. 2; Advantec Toyo Kaisha Ltd., Tokyo, Japan) in a 3 cm Petri dish. MeOH was evaporated in a draft chamber. Then, the filter paper in the Petri dishes was moistened with 0.8 mL of a 0.05% (*v*/*v*) aqueous solution of Tween 20. After germination in the darkness at 25 °C for 24 h, 10 germinated seeds of mustard green (*Brassica juncea*) were then placed into the Petri dishes. The length of their shoots and roots was measured after 48 h of incubation in the darkness at 25 °C. MeOH (0.2 mL) was added to the filter paper in the Petri dish of the control treatment and evaporated as described above. Control seeds were then placed into filter paper moistened with the aqueous solution of Tween 20 without the extract. The bioassay was repeated five times using a randomized design with 10 plants for each determination [11].

## 4. Conclusions

Allelopathic activity of MeOH extracts from nine OM rice varieties (2395, 3536, 4498, 5451, 5930, 6976, 7347, N406, and 380) on shoot and root length of barnyardgrass showed that the MeOH extract of OM 5930 posed higher allelopathic activity than others, and this indicated that the OM 5930 may be priority selected for research program on isolating allelochemicals in rice.

Compared to previous works, our study is the first to report the content of a variety of phenolic compounds in rice of different rice cultivars. The developed HPLC-MS/MS method, combined with data analysis based on the online XCMS platform, provides the sensitivity and selectivity needed to quickly and accurately quantify phenolic compounds as allelochemicals in an organic rich matrix and plant materials. Twenty allelochemicals were detected in nine OM rice cultivars, namely: 2,4-dihydroxybenzaldehyde, 2,6-dimethoxybenzoic acid, 3,4-dihydroxybenzoic acid, 3,4-dihydroxyphenylacetic acid, 3-hydroxybenzoic acid, 4-hydroxybenzoic acid, 5-methoxysalicylic acid, 7-oxostigmasterol, benzoic acid, 2,4-dimethoxybenzoic acid, 2,5-dihydroxybenzoic acid, 3,4-dimethoxybenzoic acid, 3,5-dihydroxybenzoic acid, 3,5-dimethoxybenzoic acid, cinnamic acid, coumarin, ergosterol peroxide, *p*-hydroxycinnamic acid, salicylic acid, and vanillic acid. Among these, 3,4-dimethoxybenzoic acid, 3,5-dimethoxybenzoic acid, 3,5-dihydroxybenzoic acid, 2,4-dihydroxybenzaldehyde, 2,6-dimethoxybenzoic acid, 5-methoxysalicylic acid, 2,4-dimethoxybenzoic acid, and coumarin have not been reported as allelochemicals previously in rice yet. The OM 5930 has the highest concentration of allelochemicals and the lowest concentration of stimulant (allantoin) as compared to other cultivars. Herein, our study initiates the investigation and semi-quantification of previously identified allelochemicals in rice plants capable of inhibiting weeds in rice fields, particularly on barnyardgrass. Future studies should be focused on UHPLC-MS/MS analysis of allelochemicals in OM rice cultivars.

Five feature allelochemicals were identified and quantified in 100 g of OM 5930 fresh rice by using bioassay-guided fractionation and purification. They are salicylic acid (5.0076 mg), vanillic acid (0.1246 mg), *p*-coumaric acid (0.1590 mg), 2,4-dimethoxybenzoic acid (0.1045 mg), and cinnamic acid (3.3230 mg). These compounds were active at concentrations greater than 0.5 mM and the average EC_50_ were 1.24 mM. The physical presence and allelopathic activities of the five above allelochemicals qualified in OM 5930 rice cultivar provided additional biological evidence in support of the results inferred from the analysis and identification of previously-published allelochemicals in the nine OM rice cultivars, especially, OM 5930. The result is also explored to provide insights into the biological activities of those five allelochemicals being studied and highlighted their role in plant growth inhibitory process, hence, delivering to biological understanding of the important role of OM 5930 rice cultivar in biological control of weed and its application in the sustainable ecology system.

## Figures and Tables

**Table 1:** Effect of MeOH extracts from nine OM rice cultivars on the growth of barnyardgrass shoot.

Concentrationg mL^−1^	Inhibition (%)								
	OM 2395	OM 3536	OM 4498	OM 5451	OM 5930	OM 6976	OM 7347	OM 380	OM N406
Control	0.00b ± 0.73	0.00b ± 0.50	0.00a ± 0.40	0.00a ± 0.60	0.00a ± 0.20	0.00a ± 0.40	0.00a ± 0.30	0.00a ± 0.28	0.00a ± 0.70
0.01	−4.50a ± 0.85	−4.23a ± 0.62	0.16a ± 0.67	9.54b ± 0.67	5.60b ± 0.51	7.42b ± 0.46	2.44a ± 0.48	12.2ab ± 0.55	0.96a ± 0.54
0.03	0.29b ± 0.78	2.45b ± 0.77	6.36b ± 0.70	12.30c ± 0.56	12.42c ± 0.50	19.17c ± 0.56	8.99b ± 0.31	17.3b ± 0.63	15.85b ± 1.33
0.1	21.00c ± 0.79	22.14c ± 1.02	8.65b ± 0.95	17.13d ± 0.59	20.83d ± 0.83	24.66d ± 0.73	10.42b ± 0.43	18.2b ± 0.44	18.98c ± 0.73
0.3	57.11d ± 0.79	38.36d ± 1.21	32.98c ± 1.02	18.71d ± 0.65	57.39e ± 0.40	27.98e ± 0.52	12.13c ± 0.50	38.5c ± 0.81	25.15d ± 0.49
0.5	64.58e ± 0.27	55.01e ± 0.90	54.01d ± 1.05	26.03e ± 0.49	64.96f ± 0.53	50.58f ± 0.57	23.08d ± 0.57	45.3c ± 0.56	37.59e ± 0.56
1.0	76.22f ± 0.42	61.02f ± 1.16	90.75e ± 0.15	72.12f ± 0.37	98.77g ± 0.09	87.17g ± 0.23	30.77e ± 0.54	60.3d ± 0.13	54.83f ± 1.05
F	**	**	**	**	**	**	**	**	**
C.V. (%)	1.73	1.92	1.95	1.86	0.82	1.86	1.06	0.79	0.81

C.V. is coefficient of variation. OM is appreviated of O Mon, a location where rice cultivar was bred. Values in the same column followed by one or more identical letters are not statistically significant in the Duncan test. ** difference at 1% significance level. Negative values represent the stimulation of the extract on the root length of barnyardgrass.

**Table 2:** Effect of MeOH extracts from nine OM rice cultivars on the growth of barnyardgrass root.

Concentrationg mL^−1^	Inhibition (%)								
	OM 2395	OM 3536	OM 4498	OM 5451	OM 5930	OM 6976	OM 7347	OM 380	OM N406
Control	0.00a ± 0.79	0.00b ± 0.80	0.00a ± 0.80	0.00a ± 0.60	0.00a ± 0.40	0.00b ± 0.80	0.00a ± 0.70	0.00a ± 0.93	0.00a ± 0.80
0.01	21.27b ± 0.63	−10.44a ± 0.83	6.74b ± 1.00	0.58a ± 0.67	11.76b ± 0.83	−19.62a ± 0.48	12.70b ± 0.68	12.8b ± 0.81	4.63b ± 0.75
0.03	30.48c ± 0.48	1.50b ± 0.85	22.63c ± 0.82	16.26b ± 0.81	29.29c ± 0.90	−17.93a ± 0.54	28.24c ± 0.63	28.5bc ± 0.11	12.20c ± 0.63
0.1	32.46d ± 0.60	27.70c ± 1.36	44.05d ± 0.85	22.87c ± 0.60	49.71d ± 1.1	16.52c ± 0.89	30.64d ± 0.64	38.1c ± 0.96	18.74d ± 1.11
0.3	46.09e ± 0.84	46.67d ± 0.94	74.58e ± 0.86	42.48d ± 0.49	66.93e ± 0.80	24.13d ± 0.37	32.71e ± 0.51	45.2d ± 0.75	19.53d ± 0.68
0.5	57.65f ± 0.62	56.09e ± 0.46	88.12f ± 0.42	51.95e ± 0.56	85.67f ± 0.64	35.41e ± 0.72	34.40f ± 0.65	49.4d ± 0.39	30.19e ± 0.96
1.0	78.03g ± 0.40	68.05f ± 0.43	92.83g ± 0.12	78.05f ± 0.60	99.39g ± 0.05	86.56f ± 0.12	50.30g ± 0.84	56.6e ± 0.14	69.63f ± 1.06
F	**	**	**	**	**	**	**	**	**
CV (%)	1.39	1.82	1.18	1.93	0.76	1.81	1.02	1.25	1.03

C.V. is coefficient of variation. OM is appreviated of O Mon, a location where rice cultivar was bred. Values in the same column followed by one or more identical letters are not statistically significant in the Duncan test. ** difference at 1% significance level. Negative values represent the stimulation of the extract on the root length of barnyardgrass.

**Table 3:** A list of allelochemicals detected in 9 rice cultivars.

No.	Allelochemicals *	Cultivars (*Oryza sativa* L.) **									Detection
		*1*	*2*	*3*	*4*	*5*	*6*	*7*	*8*	*9*	Frequency (%)
1	2,4-dihydroxybenzaldehyde				√						11.1
2	2,6-dimethoxybenzoic acid		√					√			22.2
3	3,4-dihydroxybenzoic acid			√	√	√		√	√		55.6
4	3,4-dihydroxyphenylacetic acid			√		√			√		33.3
5	3-hydroxybenzoic acid	√			√	√	√	√	√		66.7
6	4-hydroxybenzoic acid	√			√	√	√	√	√		66.7
7	5-methoxysalicylic acid			√							11.1
8	7-oxostigmasterol	√	√			√	√	√	√	√	77.8
9	Benzoic acid			√							11.1
10	2,4-dimethoxybenzoic acid	√	√			√	√	√	√		66.7
11	2,5-dihydroxybenzoic acid			√	√	√		√	√		55.6
12	3,4-dimethoxybenzoic acid	√	√			√	√	√			55.6
13	3,5-dihydroxybenzoic acid			√	√	√		√	√		55.6
14	3,5-dimethoxybenzoic acid	√	√			√	√	√			55.6
15	Cinnamic acid	√	√	√	√	√	√	√	√		88.9
16	Coumarin			√							11.1
17	Ergosterol peroxide								√		11.1
18	*p*-hydroxycinnamic acid	√	√	√	√	√	√	√	√	√	100.0
19	Salicylic acid	√			√	√	√	√	√		66.7
20	Vanillic acid			√		√			√		33.3
	Total	9	7	10	9	14	9	13	13	2	
	Percentage (%)	45	35	50	45	70	45	65	65	10	

* Allelochemicals putatively identified using a mass spectral library (METLIN) denote the identification level of 2. ** OM 5451 (1), OM 380 (2), OM 3536 (3), OM 6976 (4), OM 4498 (5), OM 2395 (6), OM N406 (7), OM 5930 (8), and OM 7347 (9). √ means the allelochemical existed in the associated rice cultivar.

**Table 4:** Molecular information of 8 predominant allelochemicals detected in 9 OM rice cultivars by ultra-high-performance liquid chromatography mass spectrometry (UHPLC/MS) *.

No.	Allelochemicals **	Formula	Molecular Ion	Theoretical Mass	Observed Mass	Mass Error
1	Allantoin	C_4_H_6_N_4_O_3_	[M + H]^+^	159.0512	159.0513	0.63
2	Benzoic acid	C_7_H_6_O_2_	[M + H]^+^	123.0441	123.0441	0.81
3	Cinnamic acid	C_9_H_8_O_2_	[M + H]^+^	149.0597	149.0596	−0.67
4	Vanillic acid	C_8_H_8_O_4_	[M + H]^+^	169.0495	169.0496	0.59
5	Coumarin	C_9_H_6_O_2_	[M + H]^+^	147.0441	147.0440	−0.68
6	Salicylic acid	C_7_H_6_O_3_	[M-H]^-^	137.0245	137.0244	0.73
7	7-oxostigmasterol	C_29_H_46_O_2_	[M + H-H_2_O]^+^	409.3465	409.3471	1.46
8	Ergosterol peroxide	C_28_H_44_O_3_	[M + H-H_2_O]^+^	411.3258	411.3269	2.67

* UHPLC-MS analysis was performed on a Bruker maXis impact quadrupole-time-of-flight mass spectrometer coupled with a Waters ACQUITY UPLC system. ** The identification level is 2.

**Table 5:** Relative abundance of predominant allelochemicals in 9 tested rice cultivars.

OM Cultivar	Predominant Allelochemicals in 9 Cultivars *							
	Cinnamic Acid	Vanillic Acid	Coumarin	Benzoic Acid	7-Oxostigmasterol	Ergosterol Peroxide	Allantoin	Salicylic Acid
5451	13.94 ab	1.07 c	8.44 a	7.35 ab	2.04 cd	-	9.16 a	17.56 a
380	11.24 cd	1.32 c	6.23 bc	5.41 c	1.62 d	-	5.39 b	6.67 e
3536	10.83 d	1.33 b	6.98 b	4.75 d	2.25 c	-	4.27 b	12.07 bcd
6976	14.19 a	-	5.93 bc	8.26 a	1.31 e	-	-	10.92 cde
4498	12.55 bc	1.64 a	7.75 b	-	2.00 cd	-	4.96 b	19.54 a
2395	11.89 cd	-	7.25b	6.36 bc	3.06 b	-	4.68 b	14.76 abc
N406	12.99 abc	-	7.25 b	5.98 c	3.32 b	-	2.84 c	16.94 ab
5930	14.11 ab	1.33 b	8.23 a	5.89 c	4.85 a	2.36	-	17.06 ab
7347	-	-	4.65 c	-	1.51 de	-	3.81bc	8.20 de

* Relative abundance of predominant allelochemicals detected by XCMS online platform. Different characters in the same column (same compound) show a statistically significant difference between cultivars at *p* < 0.05.

**Table 6:** Analysis results of allelochemicals in OM 5930 * rice cultivar by HPLC.

No.	Allelochemicals **	Retention	Purity	Allelochemical Content	
		Time (min.)	(%)	In Rice Extract (mg mL^−1^)	In 1g Fresh Rice (mg g^−1^)
1.	Salicylic acid	11.469	98.9	0.7715	0.0501
2.	Vanillic acid	11.126	99.7	0.0192	0.0012
3.	*p*-Coumaric acid	20.269	99.7	0.0245	0.0016
4.	2,4-dimethoxybenzoic acid	30.058	99.7	0.0161	0.0010
5.	Cinnamic acid	29.902	98.7	0.5210	0.0333

* The volume of OM 5930 extract (V = 4.5 mL) corresponds to 69.33 g of fresh rice plants remaining after C18 SPE operation; 1 mL of extract contains 15.41 g of fresh rice plants. ** The identification level is 1.

## Supplementary Materials

The following are available online at https://www.mdpi.com/2218-1989/10/6/244/s1, Table S1: Major previously-reported allelochemicals in *Oryza sativa* L. species, Table S2: Allelochemicals effecting pests detected in 9 OM rice cultivars by XCMS platform.

## References

[1] Cosslett T., Cosslett P. (2018). Rice Trade of the Mainland Southeast Asian Countries: Cambodia, Laos, Thailand, and Vietnam. Sustainable Development of Rice and Water Resources in Mainland Southeast Asia and Mekong River Basin.

[2] Khai N., Tinh T., Tin H., NV S. (2018). Reducing Greenhouse Gas Emissions in Rice Grown in the Mekong Delta of Vietnam. Environ. Pollut. Clim. Chang..

[3] Chauhan B. (2013). Strategies to manage weedy rice in Asia. Crop. Prot..

[4] Smith R. (1988). Weed Thresholds in Southern U.S. rice, *Oryza sativa*. Weed. Technol..

[5] Chin D. (2001). Biology and management of *Barnyardgrass,* red *Sprangletop* and weedy rice. Weed Biol. Manag..

[6] Rimando A., Olofsdotter M., Dayan F., Duke S. (2001). Searching for Rice Allelochemicals. Agron. J..

[7] Khanh T., Xuan T., Chin D., Chung I., Elzaawely A., Tawata S. (2006). Current status of biological control of paddy weeds in Vietnam. Weed Biol. Manag..

[8] Khanh T., Xuan T., Chung I. (2007). Rice allelopathy and the possibility for weed management. Ann. Appl. Biol..

[9] Amb M., Ahluwalia A. (2016). Allelopathy: Potential Role to Achieve New Milestones in Rice Cultivation. Rice Sci..

[10] Thi H., Lin C., Smeda R., Fritschi F. (2014). Isolation and purification of growth-inhibitors from Vietnamese rice cultivars: Growth-inhibitors from Vietnamese rice cultivars. Weed Biol. Manag..

[11] Thi H., Lin C., Smeda R., Leigh N., Wycoff W., Fritschi F. (2014). Isolation and identification of an allelopathic phenylethylamine in rice. Phytochemistry.

[12] Thi H.L., Zhou H., Lin C.-H., Liu S., Berezin M.Y., Smeda R.J., Fritschi F.B. (2017). Synthesis and plant growth inhibitory activity of N-trans-cinnamoyltyramine: Its possible inhibition mechanisms and biosynthesis pathway. J. Plant Interact..

[13] Olofsdotter M. (1998). Allelopathy in Rice.

[14] Salam M., Kato-Noguchi H. (2009). Screening of Allelopathic Potential Bangladesh Rice Cultivars by Donor-Receiver Bioassay. Asian J. Plant Sci..

[15] Olofsdotter M., Rebulanan M., Madrid A., Dali W., Navarez D., Olf D.C. (2002). Why phenolic acids are unlikely primary allelochemicals in rice. J. Chem. Ecol..

[16] Moore W., Meyers D.A., Wenzel S., Teague W., Li H., Li X., D’Agostino R., Castro M., Curran-Everett D., Fitzpatrick A.M. (2010). Identification of asthma phenotypes using cluster analysis in the severe asthma research program. Am. J. Respir. Crit. Care Med..

[17] Sumner L.W., Amberg A., Barrett D., Beale M.H., Beger R., Daykin C.A., Fan T.W.-M., Fiehn O., Goodacre R., Griffin J.L. (2007). Proposed minimum reporting standards for chemical analysis. Metabolomics.

[18] Shindo H., Ohta S., Kuwatsuka S. (1978). Behavior of phenolic substances in the decaying process of plants. Soil Sci. Plant Nutr..

[19] Macías F., Chinchilla N., Varela R., Molinillo J. (2006). Bioactive steroids from *Oryza sativa* L.. Steroids.

[20] Bouillant M., Jacoud C., Zanella I., Favre-Bonvin J., Bally R. (1994). Identification of 5-(12-heptadecenyl)-resorcinol in rice root exudates. Phytochemistry.

[21] Seal A., Haig T., Pratley J. (2004). Evaluation of putative allelochemicals in rice root exudates for their role in the suppression of arrowhead root growth. J. Chem. Ecol..

[22] Chou C., Lin H. (1976). Autointoxication mechanism of *Oryza sativa* I. Phytotoxic effects of decomposing rice residues in soil. J. Chem. Ecol..

[23] Li Z., Wang Q., Ruan X., Pan C., Jiang D. (2010). Phenolics and plant allelopathy. Molecules.

[24] Chou C., Chang F., HI O. (1991). Allelopathic potentials of a wild rice, *Oryza Perennis*. Taiwania.

[25] Chung I., Kim K., Ahn J., Chun S., Kim C., Kim J., Kim S. (2002). Screening of allelochemicals on barnyardgrass (*Echinochloa crus-galli*) and identification of potentially allelopathic compounds from rice (*Oryza sativa*) variety hull extracts. Crop. Prot..

[26] Mattice J., Lavy T., Skulman B., Dilday R. (1998). Searching for allelochemicals in rice that control ducksalad. Allelopathy in Rice.

[27] Kato-Noguchi H. (2008). Allelochemicals released from rice plants. Jpn. J. Plant Sci..

[28] Chou C., Patrick Z. (1976). Identification and phytotoxic activity of compounds produced during decomposition of corn and rye residues in soil. J. Chem. Ecol..

[29] Fernandez C., Lelong B., Vila B., Mévy J., Robles C., Greff S., Dupouyet S., Bousquet-Mélou A. (2006). Potential allelopathic effect of *Pinus halepensis* in the secondary succession: An experimental approach. Chemoecology.

[30] Zhou B., Kong C., Li Y., Wang P., Xu X. (2013). Crabgrass (*Digitaria sanguinalis*) allelochemicals that interfere with crop growth and the soil microbial community. J. Agric. Food Chem..

[31] Dornbos D., Spencer G. (1990). Natural products phytotoxicity A bioassay suitable for small quantities of slightly water-soluble compounds. J. Chem. Ecol..

[32] Yamamoto Y. (2009). Movement of allelopathic compound coumarin from plant residue of sweet vernalgrass (*Anthoxanthum odoratum* L.) to soil. Grassl. Sci..

[33] Chon S., Kim Y., Lee J. (2003). Herbicidal potential and quantification of causative allelochemicals from several compositae weeds. Weed Res..

[34] Chon S., Kim Y. (2004). Herbicidal potential and quantification of suspected allelochemicals from four grass crop extracts. J. Agron. Crop. Sci..

[35] Takemura T., Kamo T., Sakuno E., Hiradate S. (2013). Discovery of coumarin as the predominant allelochemical in *Guricidia sepium*. J. Trop. Sci..

[36] Choi G.-H., Ro J.-H., Park B.-J., Lee D.-Y., Cheong M.-S., Lee D.-Y., Seo W.-D., Kim J.H. (2016). Benzaldehyde as a new class plant growth regulator on *Brassica campestris*. J. Appl. Biol. Chem..

[37] Wang P., Kong C.-H., Hu F., Xu X. (2007). Allantoin involved in species interactions with rice and other organisms in paddy soil. Plant Soil.

[38] Hegab M., Khodary S., Hammouda O., Abdelgawad H. (2008). Autotoxicity of chard and its allelopathic potentiality on germination and some metabolic activities associated with growth of wheat seedlings. Afr. J. Biotechnol..

[39] Yu J., Matsui Y. (1997). Effects of root exudates of cucumber (*Cucumis sativus*) and allelochemicals on ion uptake by cucumber seedlings. J. Chem. Ecol..

[40] Einhellig F., Rasmussen J. (1979). Effects of three phenolic acids on chlorophyll content and growth of soybean and grain sorghum seedlings. J. Chem. Ecol..

[41] Reigosa M., Sánchez-Moreiras A., González L. (1999). Ecophysiological approach in allelopathy. Crit. Rev. Plant Sci..

[42] Duke S.I. (2003). Ecophysiological aspects of allelopathy. Planta.

[43] Weir T., Park S., Vivanco J. (2004). Biochemical and physiological mechanisms mediated by allelochemicals. Curr. Opin. Plant Biol..

[44] Wagner A., Donaldson L., Ralph J., Jouanin L., Lapierre C. (2012). Chapter 2—Lignification and lignin manipulations in conifers. Advances in Botanical Research.

[45] Baleroni C., Ferrarese M., Souza N., Ferrarese-Filho O. (2000). Lipid accumulation during canola seed germination in response to cinnamic acid derivatives. Biol. Plant.

[46] Ding J., Sun Y., Xiao C., Shi K., Zhou Y., Yu J. (2007). Physiological basis of different allelopathic reactions of cucumber and figleaf gourd plants to cinnamic acid. J. Exp. Bot..

[47] Politycka B., Mielcarz B. (2007). Involvement of ethylene in growth inhibition of cucumber roots by ferulic and p-coumaric acids. Allelopath. J..

[48] Roger M., Malvido-Pazos E. (2007). Phytotoxic effects of 21 plant secondary metabolites on arabidopsis thaliana germination and root growth. J. Chem. Ecol..

[49] Salvador A., Cavaleiro A., Sousa D., Alves M., Pereira M. (2013). Endurance of methanogenic *Archaea* in anaerobic bioreactors treating oleate-based wastewater. Appl. Microbiol. Biotechnol..

[50] Bacilio-Jimenez M., Aguilar-Flores S., Ventura-Zapata E., Perez E., Bouquelet S., Zenteno E. (2003). Chemical characterization of root exudates from rice (*Oryza sativa*) and their effects on the chemotactic response of endophytic bacteria. Plant Soil.

[51] Kato-Noguchi H., Ino T., Sata N., Yamamura S. (2002). Isolation and identification of a potent allelopathic substance in rice root exudates. Physiol. Plant..

[52] Kato-Noguchi H., Ino T. (2003). Rice seedlings release momilactone B into the environment. Phytochemistry.

[53] Kong J., Liang N.H., Xu F.K., Hu F., Wang P., Jiang Y. (2004). Release and Activity of Allelochemicals from Allelopathic Rice Seedlings. J. Agric. Food Chem..

