# Supplementary material for: Allelopathic Potential of Rice and Identification of Published Allelochemicals by Cloud-Based Metabolomics Platform
Source: Metabolites. 2020 Jun 15;10(6):244. doi: 10.3390/metabo10060244 (PMC7344986; doi:10.3390/metabo10060244)
Supplement: Supplementary file 1 [file metabolites-10-00244-s001.pdf]

Allelopathic potential of rice and identification of published allelochemicals by cloud-based metabolomics platform

Ho Le Thi[1,*], Nguyen Thi Cam Tu[2], Danh C. Vu[3], Nguyen Y Nhu[4], Nguyen Thi Thu Trang[5], Phong Ngoc Hai Trieu[1], Nguyen The Cuong[1], Chung-Ho Lin[4], Zhentian Lei[6], Lloyd W. Sumner[6,7], Le Van Vang[2]

[1]Cuu Long Delta Rice Research Institute, Can Tho, Vietnam

[2]College of Agriculture and Applied Biosciences – Can Tho University, Vietnam

[3]Faculty of Technology, Van Lang University, Ho Chi Minh City, Vietnam

[4]Center for Agroforestry, School of Natural Resources, University of Missouri, Columbia, MO, USA

[5]Cultivation and Plant Protection Station. Thoi Lai District, Can Tho City

[6]Metabolomics Center, University of Missouri, Columbia, MO, USA

[7]Department of Biochemistry, Bond Life Sciences Center, University of Missouri, Columbia, MO, USA

*Corresponding author:

Phone: (+84) 944 376 329

Email: thihl.clrri@mard.gov.vn

Table S1. Major previously-reported allelochemicals in *Oryza sativa* L. species

No.	Identified chemical	Organ	Reference
1	1,2-benzenedicarboxylic acid, bis (2-ethylhexyl) ester	Root	Rimando *et al.* (2001)
2	12-methyl-tetradecanoic acid, methyl ester	Root	Kim and Kim (2000)
3	12-octadecenoic acid, methyl ester	Root	Kim and Kim (2000)
4	1-eicosanol	Root	Kim and Kim (2000)
5	1-ethyl-3,5-dimethylbenzene	Root	Kim and Kim (2000)
6	1H-indole-3-carboxaldehyde	Root	Rimando *et al.* (2001)
7	1H-indole-3-carboxylic acid	Root	Rimando *et al.* (2001)
8	1H-indole-5-carboxylic acid	Root	Rimando *et al.* (2001)
9	1-phenyl-2-hydroxy-3,7-dimethyl-11-aldehydic-tetradecane-2-β-D-glucopyranoside	Stem	Chung *et al.* (2006a)
10	1-tetratriacontanol	Root	Bouillant *et al.* (1994)
11	2- hydroxyphenylacetic acid	Root	Seal *et al.* (2004b),<br>Kim and Kim (2000)
12	2-methyl-1,4-benzenediol	Root	Kim and Kim (2000)
13	3,4-dihydroxyhydrocinnamic acid	Root	Rimando *et al.* (2001)
14	3-epicosene (3-eicosene)	Root	Kim and Kim (2000)
15	3-hydroxy-4-methoxybenzoic acid (isovanillic acid)	Root	Mattice *et al.* (1998); Rimando *et al.* (2001)
16	3-hydroxybenzoic acid	Root	Mattice *et al.* (1998)
17	3-hydroxy-β-ionone	Whole plant	Kato-Noguchi *et al.* (2011)
18	3-isopropyl-5-acetoxycyclohexene-2-one-1 (Cyclohexenone)	Root	Kong *et al.* (2004a)
19	4-ethylbenzaldehyde	Root	Mattice *et al.* (1998),<br>Kim and Kim (2000)
20	4-hydroxybenzaldehyde	Root	Mattice *et al.* (1998)
21	4-hydroxybenzoic acid	Root	Mattice *et al.* (1998)
22	4-hydroxycinnamic acid	Root	Rimando *et al.* (2001)
23	4-hydroxyhydrocinamic acid	Root	Rimando *et al.* (2001)
24	4-hydroxyphenylacetic acid	Root	Bouillant *et al.* (1994)
25	4-phenylbutyric acid	Root	Seal *et al.* (2004b); Bouillant *et al.* (1994)
26	4-vinylphenol	Whole plant	Song *et al.* (2004)
27	5-(12-heptadecenyl)-resorcinol (Alkyl resorcinol)	Root	Bouillant *et al.* (1994)

28	5,4′-dihydroxy-3′,5′-dimethoxy-7-O-β-glucopyranosylflavone	Root	Kong *et al.* (2004a, b)
29	4',5-dihydroxy-7-methoxyflavanone	Seedling	Kato-Noguchi *et al.* (2010)
30	4′,5,7-trihydroxy-3′,5′-dimethoxyflavone	Root	Kong *et al.* (2004a)
31	5-hydroxyindole-2-carboxylic acid	Whole plant	Song *et al.* (2004)
32	5-hydroxyindole-3-acetic acid	Root	Seal *et al.* (2004b); Bouillant *et al.* (1994)
33	7-hexadecenoic acid, methyl ester	Root	Kim and Kim (2000)
34	7-oxo-stigmasterol	Fresh root, leaf and stem	Macias *et al.* (2006)
35	9,12-octadecadienoic acid	Root	Kim and Kim (2000)
36	9-hydroxy-4-megastigmen-3-one	Whole plant	Kato-Noguchi *et al.* (2011)
37	Abietic acid	Root	Seal *et al.* (2004b)
38	Aliphatic acids	Root	Kong *et al.* (2004a)
39	Alkaloid A (dẫn xuất của Alkaloids)	Seedling incubated in dark condition	Suzuki *et al.* (1996)
40	Alkylresorcinols	Husk and root	Suzuki *et al.*, 1996; 1998
41	Azelaic acid	Root	Rimando *et al.* (2001)
42	Benzoic acid	Root	Kuwatsuka and Shindo (1973)
43	Blumenal A	Root	Kato-Noguchi *et al.* (2012)
44	Caffeic acid	Husk	Kuwatsuka and Shind (1973)
		Root	Seal *et al.* (2004a)
45	Cholest-5-en-3(β)-ol	Root	Kim and Kim (2000)
46	Cinnamic aldehyde	Root	Kim and Kim (2000)
47	*cis*-1-butyl-2-methylcyclopropane	Root	Kim and Kim (2000)
48	Cytokinin	Root	Soejima *et al.* (1992)
49	Dehydroabietic acid	Root	Kim and Kim (2000)
50	Dicyclohexyl orizane	Root	Bouillant *et al.* (1994)
51	Diterpenoids	Whole plant	
52	Ergosterol peroxide	Fresh root, leaf and stem	Macias *et al.* (2006)
53	Ethyl-4-(o-nitrophenyl)-3-thioallophanate	Husk and root	Kong *et al.* (2002b)
54	Ethyl-4-(o-nitrophenyl)-3-allophanate	Husk and root	Kong *et al.* (2002b)
55	Ferulic acid	Decomposed husk	Chou & Lin (1976)
		Leaf and stem	Chou *et al.* (1991)
56	Flavonoid	Root	Kong *et al.* (2004a)
57	Gallic acid	Leaf and stem	Chou *et al.* (1991)
58	Gentisic acid	Husk	Kuwatsuka and Shindo (1973)
59	Grasshopper ketone	Root	Kato-Noguchi *et al.*

			(2012)
60	Indole-5-carboxylic acid	Root	Seal *et al.* (2004b), Bouillant *et al.* (1994)
61	Lanast-7,9(11)-dien3α,15α-diol-3α-D-glucofuranoside	Stem	Chung *et al.* (2006a)
62	Mandelic acid	Decomposed husk	Chou and Lin (1976)
63	*m*-coumaric acid	Leaf and stem	Chou *et al.* (1991)
64	Mercaptoacetic acid	Whole plant	Song *et al.* (2004)
65	Momilactone A	Whole plant	Kato-Noguchi (2008b)
66	Momilactone B	Stem	Cartwright *et al.* (1981), Chung *et al.* (2005a)
		Leaf and husk	Kato *et al.* (1973)
		Root	Kato-Noguchi and Ino (2003, 2005)
67	*N-trans*-cinnamoyltyramine	Whole plant (OM5930)	Ho Le Thi *et al.* (2014)
68	*o*-coumaric acid	Leaf and stem	Chou *et al.* (1991)
69	Octadecane	Root	Kim and Kim (2000)
70	*o*-hydroxyphenylacetic acid	Root	Bouillant *et al.* (1994)
71	Oryxalexins (A, B, C, D, E và F)	Whole plant	Kong *et al.* (2002b)
		Whole plant	Kato *et al.*, (1977, 1995)
72	*p*-coumaric acid	Decomposed husk	Chou and Lin (1976)
		Leaf and stem	Chou and Chiou (1979)
		Rice soil	Rimando *et al.* (2001)
		Root	Seal *et al.* (2004a)
73	Phenolic aldehyde	Root	Kong *et al.* (2004a)
74	Phenolic acids	Root	Kuwatsuka and Shindo (1973)
75	*p*-hydroxybenzoic acid	Leaf and stem	Chou *et al.* (1991)
		Rice soil	Olofsdotter *et al.* (2002b)
		Root	Seal *et al.* (2004a)
76	Phytocassanes (A, B, C và D)	Whole plant	Koga *et al.* (1995)
77	Protocatechuic acid	Husk	Kuwatsuka and Shindo (1973)
		Leaf and husk	Chou *et al.* (1991)
78	*p*-Salicylic acid	Decomposed husk	Chou and Lin (1976)
79	Resorcinols	Root	Bouillant *et al.* (1994)
80	Salicyclic acid	Husk	Kuwatsuka and Shindo (1973)
		Root	Seal *et al.* (2004a)
81	Sinapic acid	Husk	Kuwatsuka and Shindo (1973)
82	Stearic acid	Rice soil	Mattice *et al.* (1998)

83	Stigmastanols (-3β-*p*-glyceroxydihydrocoumaroate and -3β-*p*- butanoxyd-ihydrocoumaroate)	Stem	Chung *et al.* (2006a)
84	Syringic acid	Husk	Kuwatsuka and Shindo (1973)
		Rice soil	Chou and Chiou (1979)
		Decomposed husk	Chou *et al.* (1977)
		Root	Seal *et al.* (2004a)
85	*t*-Coumaric acid	Root	Seal *et al.* (2004a)
		Husk	Kuwatsuka and Shindo (1973)
86	Tetradecanoic acid	Root	Mattice *et al.* (1998)
87	*t*-Ferulic acid	Root	Seal *et al.* (2004a)
		Decomposed husk	Chou and Lin (1976)
88	*trans*-Ferulic acid	Whole plant	Song *et al.* (2004)
89	Valeric acid	Root	Mattice *et al.* (1998)
		Decomposed husk	Chou and Lin (1976)
		Rice soil	Mattice *et al.* (1998)
		Root	Seal *et al.* (2004a)
90	*β*-resorcylic acid	Husk and root	Kuwatsuka and Shindo (1973)
91	*β*-sitosterol-3-O-*β*-*D*-glucoside	Root	Bouillant *et al.* (1994)

Table S2. Allelochemicals* having effects on controlling/ stimulating pests detected in 9 OM rice cultivars by XCMS online in pair-wise job mode

CHEMICALS IN OM 4498			
**No.**	**NAME**	**ADDUCT**	**METLIN ID**
1.	(R)(-)-Allantoin	M+H	63395
2.	(S)(+)-Allantoin	M+H	63392
3.	2-(Acetyloxy)-3,5-dichlorobenzoic acid	M+H	861630
4.	2,4-Dihydroxybenzoic acid	M-H	283902
5.	2,5-Bis(trifluoromethyl)hydrocinnamic acid	M+H	492329
6.	2-amino-3,5-dihydroxybenzoic acid	M+H	330220
7.	2-Ethyl-6-hydroxybenzoic acid	M+H	276962
8.	2-Hydroxy-6-methoxybenzoic acid	M+H	374393
9.	3,4-Dihydroxybenzoic acid	M-H	267460
10.	3,4-Dihydroxyphenylacetic acid	M+H	383653
11.	3',5,7-Trihydroxy-4',8-dimethoxyisoflavone	M+H	302623

12.	3,5-Bis(trifluoromethyl)hydrocinnamic acid	M+H	419911
13.	3,5-Di-tert-butyl-4-hydroxycinnamic acid, (E)-	M-H	522176
14.	3,5-Di-tert-butyl-4-hydroxyhydocinnamic acid	M-H	361036
15.	3-Ethoxy-4-hydroxybenzoic acid	M+H	288553
16.	3-ethyl-2-hydroxybenzoic acid	M+H	300561
17.	3-Hydroxybenzoic acid	M-H	269175
18.	3-Nitro-4-(piperidin-1-yl)cinnamic acid	M-H	442253
19.	4-(Dichloroacetyl)-3-hydroxybenzoic acid	M+H	940137
20.	4,4'-[Propane-1,3-diylbis(oxy)]bis(2-hydroxybenzoic acid)	M+H-H2O	893932
21.	4-[(3,7-Dimethyloct-6-en-1-yl)oxy]benzoic acid	M-H	650690
22.	4-amino-2,6-dihydroxybenzoic acid	M+H	534515
23.	4-Hydroxybenzoic acid	M-H	287667
24.	4-Methoxy-(2E)-cinnamic acid	M+H	313925
25.	4-Pentadecylsalicylic acid	M+NH4	485437
26.	5a,6a-Epoxy-7E-megastigmene-3a,9e-diol 3-glucoside	M+Na	87854
27.	5a,6a-Epoxy-7E-megastigmene-3b,9e-diol 9-glucoside	M+Na	93030
28.	5-Amino-2,4-dihydroxybenzoic acid	M+H	288841
29.	6-n-Tridecylsalicylic acid	M+H	449737
30.	6-pentadecyl Salicylic Acid	M+NH4	45469
31.	7-Oxostigmasterol	M+H	86666
32.	9,13-Dihydroxy-4-megastigmen-3-one 9-glucoside	M+Na	91463
33.	Allantoic acid	M+H-H2O	343
34.	Allantoin	M+H	265134
35.	Allantoin	M+H	89
36.	Benzoic acid, 2-(allyloxy)-3,5-dipropyl-, methyl ester	M-H	378753
37.	Benzoic acid, 2,2'-[1,4-phenylenebis(carbonylimino)]bis[5-hydroxy-	M+H	493792
38.	Benzoic acid, 2,4,6-trihydroxy-	M+NH4	333307
39.	Benzoic acid, 2,4-dihydroxy-3-methyl-	M+H	350080
40.	Benzoic acid, 2,4-dihydroxy-3-methyl-, methyl ester	M+H	365361
41.	Benzoic acid, 2,4-dihydroxy-6-methyl-, methyl ester	M+H	347669
42.	Benzoic acid, 2,4-dimethoxy-	M+H	333768
43.	Benzoic acid, 2,5-dihydroxy-	M-H	337551
44.	Benzoic acid, 2,6-dichloro-4-hydroxy-3,5-dimethoxy-	M+H-H2O	450757
45.	Benzoic acid, 2-[(carboxymethyl)amino]-, monosodium salt	M+H	374048

46.	Benzoic acid, 2-decyl-6-hydroxy-	M-H	587378
47.	Benzoic acid, 2-hydroxy-, 2-propenyl ester	M+H	355726
48.	Benzoic acid, 2-hydroxy-, compd. with 3-[(2S)-1-methyl-2-pyrrolidinyl]pyridine (1:1)	M-H	386004
49.	Benzoic acid, 2-hydroxy-5-methoxy-	M+H	346384
50.	Benzoic acid, 3-(acetyloxy)-4-methoxy-	M-H	322689
51.	Benzoic acid, 3,4-dimethoxy-	M+H	333952
52.	Benzoic acid, 3,5-bis(1,1-dimethylethyl)-4-hydroxy-, ethyl ester	M-H	343827
53.	Benzoic acid, 3,5-dihydroxy-	M-H	334323
54.	Benzoic acid, 3,5-dihydroxy-, methyl ester	M+H	345179
55.	Benzoic acid, 3,5-dimethoxy-	M+H	342868
56.	Benzoic acid, 3,5-dipropyl-4-((2-methylallyl)oxy)-	M-H	378740
57.	Benzoic acid, 3,5-dipropyl-4-ethoxy-, ethyl ester	M-H	388040
58.	Benzoic acid, 4-(acetyloxy)-3-methoxy-	M-H	398467
59.	Benzoic acid, 4-(allyloxy)-3,5-dipropyl-, methyl ester	M-H	387743
60.	Benzoic acid, p-hydroxy-, decyl ester	M-H	371436
61.	beta-Ionone	M+H-H2O	269858
62.	Caffeic acid 3-O-glucuronide	M+NH4	96063
63.	Caffeic acid 3-sulfate	M-H	96064
64.	Caffeic acid 4-O-glucuronide	M+NH4	96065
65.	Caffeic acid 4-sulfate	M-H	96066
66.	Carbonic acid, ethyl ester, ester with salicylic acid	M-H	426988
67.	Cinnamic acid	M+H	386653
68.	Flavone, 4',7-dimethoxy-3,3',5-trihydroxy-	M+H	274757
69.	Flavone, 5,7-dimethoxy-3,3',4'-trihydroxy-	M+H	421136
70.	Glucosyringic acid	M-H	473754
71.	Homovanillic acid	M+H	336519
72.	Isovanillic acid	M+H	349613
73.	Phenyllactic acid	M+H	34529
74.	p-Hydroxycinnamic acid	M+H-H2O	354145
75.	Salicylic acid	M-H	287200
76.	Salicylic acid beta-D-glucoside	M+Na	95685
77.	Salicylic acid, (2-(2-(p-chlorophenoxy)-2,2-dimethylacetoxy)ethyl)ester	M-H	268358
78.	Salicylic acid, 4-(2-(diethylamino)acetamido)-, hexyl ester, hydrochloride	M+H	427261
79.	Salicylic acid, 4-(3-(benzylamino)propionamido)-, ethyl ester, monohydrochloride	M+Na	425714

80.	Salicylic acid, 4-(3-(diethylamino)propionamido)-, pentyl ester, hydrochloride	M+H	425741
81.	Salicylic acid, 4-(3-(isopropylamino)propionamido)-, hexyl ester, hydrochloride	M+H	425758
82.	Salicylic acid, 4-(3-piperidinopropionamido)-, hexyl ester, hydrochloride	M-H	425760
83.	Salicylic acid, 4-(N(sup 2)-(1,3-dihydroxy-2-(p-nitrophenyl)-2-propyl)oxamido)-, D-threo-, (-)-	M+NH4	395938
84.	Salicylic acid, 4-(N(sup 2)-(1,3-dihydroxy-2-(p-nitrophenyl)-2-propyl)oxamido)-, L-threo-, (+)-	M+NH4	395941
85.	Salicylic acid, 4-amino-, acetate, sodium salt	M+H	392378
86.	Salicylic acid, dichloroacetate	M+H	498655
87.	Salicylic acid, p-butylamino-, 1-ethyl-4-piperidyl ester, hydrochloride	M+H	509254
88.	Vanillic acid	M+H	335542

CHEMICALS IN OM 2395			
**No.**	**NAME**	**ADDUCT**	**METLIN ID**
1.	(R)(-)-Allantoin	M+H	63395
2.	(S)(+)-Allantoin	M+H	63392
3.	2,2'-(Decane-1,10-diyldiazanediyl)bis(phenylacetic acid)--hydrogen bromide (1/1)	M-H	841096
4.	2,3,5,6-Tetrafluoro-4-hydroxybenzoic acid--water (1/1)	M-H	699091
5.	2-Ethyl-6-hydroxybenzoic acid	M+H	276962
6.	3-Cyclopentyl-5-tert-butyl-4-hydroxybenzoic acid	M+H-H2O	519898
7.	3-Ethoxy-4-hydroxybenzoic acid	M+H-H2O	288553
8.	3-ethyl-2-hydroxybenzoic acid	M+H	300561
9.	3-Hexaprenyl-4,5-Dihydroxybenzoic acid	M-H	5976
10.	3-Hydroxybenzoic acid	M-H	269175
11.	4-(2-Hydroxy-3-methoxy-propoxy)-benzoic acid	M+H-H2O	580872
12.	4,4'-[Propane-1,3-diylbis(oxy)]bis(2-hydroxybenzoic acid)	M+H-H2O	893932
13.	4-Hydroxybenzoic acid	M+H-H2O	287667
14.	4-Methoxy-(2E)-cinnamic acid	M+H-H2O	313925
15.	7-Oxostigmasterol	M+H	86666
16.	8-Quinolinol salicylic acid (1:1)	M-H	311563
17.	Allantoic acid	M+H-H2O	343
18.	Allantoin	M+H	265134
19.	Allantoin	M+H	89
20.	Benzoic acid, 2-(allyloxy)-3,5-dipropyl-	M+H-H2O	378756
21.	Benzoic acid, 2-(allyloxy)-3,5-dipropyl-, methyl ester	M-H	378753
22.	Benzoic acid, 2,4-dihydroxy-3-methyl-, methyl ester	M+H-H2O	365361
23.	Benzoic acid, 2,4-dihydroxy-6-methyl-, methyl ester	M+H-H2O	347669
24.	Benzoic acid, 2,4-dimethoxy-	M+H-H2O	333768
25.	Benzoic acid, 2-[(1,3-benzodioxol-5-ylmethylene)amino]-, methyl ester	M-H	378871
26.	Benzoic acid, 2-hydroxy-, 2-propenyl ester	M+H-H2O	355726
27.	Benzoic acid, 2-hydroxy-3,4-dimethyl-	M+H	311363
28.	Benzoic acid, 2-hydroxy-6-(8-pentadecenyl)-	M+H	309832
29.	Benzoic acid, 3-(acetyloxy)-4-methoxy-	M-H	322689
30.	Benzoic acid, 3,4,5-trihydroxy-, 1,1-dimethylethyl ester	M+H-H2O	591452

31.	Benzoic acid, 3,4,5-trimethoxy-, methyl ester	M+H-H2O	344660
32.	Benzoic acid, 3,4-dimethoxy-	M+H-H2O	333952
33.	Benzoic acid, 3,5-dimethoxy-	M+H-H2O	342868
34.	Benzoic acid, 3,5-dimethoxy-4-hydroxy-, ethyl ester	M+H-H2O	502297
35.	Benzoic acid, 3,5-dipropyl-4-((2-methylallyl)oxy)-	M-H	378740
36.	Benzoic acid, 4-(8-nonenyloxy)-	M+H-H2O	485399
37.	Benzoic acid, 4-(9-acridinylamino)-3-methyl-	M-H	351647
38.	Benzoic acid, 4-(acetyloxy)-3-methoxy-	M-H	398467
39.	Benzoic acid, 4-(allyloxy)-3,5-diisopropyl-	M+H-H2O	378733
40.	Benzoic acid, 4-(allyloxy)-3,5-dipropyl-	M+H-H2O	387734
41.	Benzoic acid, 4-(allyloxy)-3,5-dipropyl-, methyl ester	M-H	387743
42.	Benzoic acid, acetylmethyl ester	M+H-H2O	358947
43.	Benzoic acid--[(2R,4R)-2-propyl-1,3-dithiolan-4-yl]methanol (1/1)	M-H	955826
44.	Benzoic acid--[(2R,4S)-2-propyl-1,3-dithiolan-4-yl]methanol (1/1)	M-H	955955
45.	Benzoic acid--[1~1~,2~1~:2~3~,3~1~-terphenyl]-1~4~,3~4~-diol (2/1)	M-H	895320
46.	Benzoic acid--4,8,11,11-tetramethylbicyclo[7.2.0]undecan-2-ol (1/1)	M+H	934910
47.	Carbonic acid, ethyl ester, ester with salicylic acid	M-H	426988
48.	Cinnamic acid	M+H	386653
49.	Dihydro-beta-ionone	M+Na	317358
50.	Dihydroferulic acid 4-O-glucuronide	M-H	96081
51.	E-Cinnamic acid	M+H	273681
52.	Homovanillic acid	M+H-H2O	336519
53.	Hydrocinnamic acid, p-((2-chloroethyl)(2-fluoroethyl)amino)-	M+NH4	504861
54.	o-Methoxyhydrocinnamic acid	M+H-H2O	24105
55.	Phenylacetic acid	M+H-H2O	269393
56.	Phenyllactic acid	M+H	34529
57.	p-Hydroxycinnamic acid	M+H-H2O	354145
58.	Salicylic acid	M+H-H2O	287200
59.	Salicylic acid beta-D-glucoside	M-H	95685
60.	Salicylic acid monoethanolamine	M+H-H2O	319803
61.	Salicylic acid, 3,5-diallyl-, methyl ester	M+H	519215
62.	Salicylic acid, 4-(2-piperidinoacetamido)-, butyl ester, hydrochloride	M+H-H2O	425724
63.	Salicylic acid, 4-(2-piperidinoacetamido)-, methyl ester, hydrochloride	M-H	426167

64.	Salicylic acid, 4-(3-(cyclohexylamino)propionamido)-, ethyl ester, monohydrochloride	M+H-H2O	425715
65.	Salicylic acid, 4-(3-piperidinopropionamido)-, hexyl ester, hydrochloride	M-H	425760
66.	Salicylic acid, 4-(3-piperidinopropionamido)-, propyl ester, hydrochloride	M+H-H2O	425719
67.	Salicylic acid, amino-, 2-hydroxyethyl ester	M+H	428715

CHEMICALS IN OM 3536			
**No.**	**NAME**	**ADDUCT**	**METLIN ID**
1.	(3,5-Difluoro-4-methoxyphenyl)acetic acid	M-H	273869
2.	(R)(-)-Allantoin	M+H	63395
3.	(S)(+)-Allantoin	M+H	63392
4.	2-(4-methylnaphthalene-1-carbonyl)benzoic acid	M-H	301669
5.	2-(difluoromethoxy)phenylacetic acid	M-H	457312
6.	2,4-Dihydroxybenzoic acid	M+H-H2O	283902
7.	2-[(2-chloro-4-methylbenzoyl)carbamothioylamino]-5-iodobenzoic acid	M-H	498198
8.	2-[3-(difluoromethoxy)phenyl]acetic Acid	M-H	457314
9.	3,4-Dihydroxybenzoic acid	M+H-H2O	267460
10.	3,4-Dihydroxyphenylacetic acid	M-H	383653
11.	3-[(2-chloro-5-iodobenzoyl)carbamothioylamino]-4-methylbenzoic acid	M-H	428446
12.	3-[(E)-({4-[Bis(2-chloroethyl)amino]-3-methoxyphenyl}methylidene)amino]benzoic acid--hydrogen chloride (1/1)	M-H	786623
13.	3-Amino-4-ethoxybenzoic acid	M-H	269132
14.	4-(Difluoromethoxy)phenylacetic acid	M-	
15.	4-(Trifluoromethyl)hydrocinnamic acid	M+H-H2O	482875
16.	4-[(E)-({4-[Bis(2-chloroethyl)amino]-3-methoxyphenyl}methylidene)amino]benzoic acid--hydrogen chloride (1/1)	M-H	786477
17.	4-Methoxy-3-(morpholin-4-yl)benzoic acid	M-H	269129
18.	5-Methoxysalicylic acid	M-H	4164
19.	6-Methyl coumarin	M+H	285280
20.	Allantoic acid	M+H-H2O	343
21.	Allantoin	M+H	265134
22.	Allantoin	M+H	89
23.	Benzoic acid	M+H	265216
24.	Benzoic acid, 2-(((6,8-dibromo-3,4-dihydro-2-(2-methoxyphenyl)-4-oxo-2-quinazolinyl)methyl)sulfonyl)-	M+NH4	402216
25.	Benzoic acid, 2-((7-chloro-4-quinolinyl)amino)-, 2-oxo-2-((1-(2-oxo-2-phenylethyl)-4-piperidinyl)oxy)ethyl ester	M-H	515902
26.	Benzoic acid, 2-(2,3-dihydro-5-methyl-2-(3-(4-(4-methyl-2-pyridinyl)-1-piperazinyl)propyl)-	M-H	396678

	3-oxo-1H-pyrazol-1-yl)-, methyl ester, trihydrochloride		
27.	Benzoic acid, 2-(5-(2-chloro-4-(trifluoromethyl)phenoxy)-2-nitrophenyl)hydrazide	M-H	391715
28.	Benzoic acid, 2-(allyloxy)-3,5-dipropyl-, methyl ester	M-H	378753
29.	Benzoic acid, 2,4-dihydroxy-3-methyl-	M-H	350080
30.	Benzoic acid, 2,5-dihydroxy-	M+H-H2O	337551
31.	Benzoic acid, 2,6-diacetamido-	M-H	399043
32.	Benzoic acid, 2-hydroxy-, compd. with 3-[(2S)-1-methyl-2-pyrrolidinyl]pyridine (1:1)	M-H	386004
33.	Benzoic acid, 2-hydroxy-5-methoxy-	M-H	346384
34.	Benzoic acid, 3,3'-(2,2,2-trichloroethylidene)bis[6-hydroxy-	M-H	416933
35.	Benzoic acid, 3,4,5-trimethoxy-, 4-(4-phenyl-1-piperazinyl)butyl ester, dihydrochloride	M-H	316390
36.	Benzoic acid, 3,4,5-tris[[(1,1-dimethylethoxy)carbonyl]oxy]-	M-H	619755
37.	Benzoic acid, 3,5-bis(acetylamino)-	M-H	354618
38.	Benzoic acid, 3,5-dihydroxy-	M+H-H2O	334323
39.	Benzoic acid, 3,5-dihydroxy-, methyl ester	M-H	345179
40.	Benzoic acid, 3,5-dipropyl-4-((2-methylallyl)oxy)-	M-H	378740
41.	Benzoic acid, 3-[3-[(2-aminoethyl)(5-isoquinolinylsulfonyl)amino]propyl]-	M-H	590150
42.	Benzoic acid, 3-amino-2,5-dimethyl-	M-H	324699
43.	Benzoic acid, 4-(((4-fluorophenoxy)acetyl)amino)-, 2-(diethylamino)ethyl ester	M-H	288765
44.	Benzoic acid, 4-((aminoiminomethyl)amino)-, 4-(methylthio)phenyl ester	M-H	509829
45.	Benzoic acid, 4-(1-carboxycyclopropyl)-2,3,5-trifluoro-, 1-ethyl ester	M-H	628972
46.	Benzoic acid, 4-(6-(1-pyrrolidinyl)-1,2,4-triazolo(3,4-a)phthalazin-3-yl)-	M+Na	516692
47.	Benzoic acid, 4-(allyloxy)-3,5-dipropyl-, methyl ester	M-H	387743
48.	Benzoic acid, 4,4'-(1,4-dioxo-1,4-butanediyl)bis-, dimethyl ester	M-H	628482
49.	Benzoic acid, 4-[(5-cyano-1-ethyl-1,6-dihydro-2-hydroxy-4-methyl-6-oxo-3-pyridinyl)azo]-, 2-(2-methoxyethoxy)ethyl ester	M-H	367347

50.	Benzoic acid, 4-chloro-2-((2-methylphenyl)amino)-, 2-(aminothioxomethyl)hydrazide	M-H	448007
51.	Benzoic acid, 4-chloro-2-((3-methylphenyl)amino)-, 2-(aminothioxomethyl)hydrazide	M-H	448009
52.	Benzoic acid, 4-chloro-2-((4-methylphenyl)amino)-, 2-(aminothioxomethyl)hydrazide	M-H	448011
53.	Benzoic acid, 4-nitro-, pentyl ester	M-H	358069
54.	Benzoic acid--[(2R,4R)-2-propyl-1,3-dithiolan-4-yl]methanol (1/1)	M-H	955826
55.	Benzoic acid--[(2R,4S)-2-propyl-1,3-dithiolan-4-yl]methanol (1/1)	M-H	955955
56.	Benzoic acid-4-13C	M+H	595330
57.	Benzoicacid,4-iodo-,ethylester	M-H	323640
58.	beta-Ionone	M+H	269858
59.	Caffeic acid 3-O-glucuronide	M+NH4	96063
60.	Caffeic acid 4-O-glucuronide	M+NH4	96065
61.	Cinnamic acid	M+H	386653
62.	Coumarin	M+H	265421
63.	Coumarin, 3-amino-4-morpholino-	M-H	317019
64.	Coumarin, 3-chloro-7-hydroxy-4-methyl-, bis(4-chlorobutyl)phosphate	M-H	430280
65.	Coumarin, 3-ethyl-	M+H	360342
66.	E-Cinnamic acid	M+H	273681
67.	Glucosyringic acid	M+Na	473754
68.	Homovanillic acid	M+H	336519
69.	Isovanillic acid	M-H	349613
70.	p-Hydroxycinnamic acid	M+H-H2O	354145
71.	Salicylic acid beta-D-glucoside	M-H	95685
72.	Salicylic acid, 3,5-diallyl-, methyl ester	M+H	519215
73.	Salicylic acid, 4-(3-piperidinopropionamido)-, hexyl ester, hydrochloride	M-H	425760
74.	Vanillic acid	M-H	335542

CHEMICALS IN OM 5930			
No.	NAME	ADDUCT	METLIN ID
1.	2,3,4-Trihydroxybenzoic acid	M+NH4	325930
2.	2,4,5-Trihydroxybenzoic acid	M+NH4	326013
3.	2,5-Bis(trifluoromethyl)hydrocinnamic acid	M+H	492329
4.	2-Ethyl-6-hydroxybenzoic acid	M+H	276962
5.	3,4-Dihydrocoumarin	M+H	265621
6.	3,4-Dihydroxyphenylacetic acid	M+H	383653
7.	3,5-Bis(trifluoromethyl)hydrocinnamic acid	M+H	419911
8.	3-ethyl-2-hydroxybenzoic acid	M+H	300561
9.	3-Hydroxybenzoic acid	M+H-H2O	269175
10.	4,4'-[1,3-Phenylenebis(carbonylazanediyl)]bis(2-hydroxybenzoic acid)	M+H	671339
11.	4,4'-[1,4-Phenylenebis(carbonylazanediyl)]bis(2-hydroxybenzoic acid)	M+H	540511
12.	4,4'-[Hexane-1,6-diylbis(oxy)]bis(2-hydroxybenzoic acid)	M+H-H2O	893933
13.	4,4'-[Propane-1,3-diylbis(oxy)]bis(2-hydroxybenzoic acid)	M+H-H2O	893932
14.	4,5-Dimethylsalicylic acid	M+H	311360
15.	4-[[4-[(4-carboxy-3-hydroxyphenyl)iminomethyl]phenyl]methylideneamino]-2-hydroxybenzoic acid	M+Na	428018
16.	4-Hydroxybenzoic acid	M+H-H2O	287667
17.	4-Methoxy-(2E)-cinnamic acid	M+H	313925
18.	7-Oxostigmasterol	M+H	86666
19.	Benzoic acid, 2-(allyloxy)-3,5-dipropyl-, methyl ester	M+H	378753
20.	Benzoic acid, 2,2'-[1,4-phenylenebis(carbonylimino)]bis-	M+Na	416785
21.	Benzoic acid, 2,2'-[1,4-phenylenebis(carbonylimino)]bis[5-hydroxy-	M+H	493792
22.	Benzoic acid, 2,4,6-trihydroxy-	M+NH4	333307
23.	Benzoic acid, 2,4-dihydroxy-3-methyl-	M+H	350080
24.	Benzoic acid, 2-[(carboxymethyl)amino]-, monosodium salt	M+H	374048
25.	Benzoic acid, 2-hydroxy-, 2-propenyl ester	M+H	355726
26.	Benzoic acid, 2-hydroxy-3,4-dimethyl-	M+H	311363
27.	Benzoic acid, 2-hydroxy-5-methoxy-	M+H	346384
28.	Benzoic acid, 2-hydroxy-6-(8-pentadecenyl)-	M+H	309832

29.	Benzoic acid, 3,3'-[1,3-phenylenebis(carbonylimino)]bis-	M+Na	364552
30.	Benzoic acid, 3,5-dihydroxy-, methyl ester	M+H	345179
31.	Benzoic acid, 3,5-dipropyl-4-((2-methylallyl)oxy)-	M+H	378740
32.	Benzoic acid, 4-(allyloxy)-3,5-dipropyl-, methyl ester	M+H	387743
33.	Benzoic acid, acetylmethyl ester	M+H	358947
34.	Benzoic acid--[(2R,4R)-2-propyl-1,3-dithiolan-4-yl]methanol (1/1)	M+Na	955826
35.	Benzoic acid--[(2R,4S)-2-propyl-1,3-dithiolan-4-yl]methanol (1/1)	M+Na	955955
36.	Benzoic acid--4,8,11,11-tetramethylbicyclo[7.2.0]undecan-2-ol (1/1)	M+H	934910
37.	beta-Ionone	M+H	269858
38.	Bis(3-nitrophenyl) methyl(phenyl)propanedioate	M+H	898560
39.	Cinnamic acid	M+H	386653
40.	Dihydro-beta-ionone	M+Na	317358
41.	E-Cinnamic acid	M+H	273681
42.	Ergosterol peroxide	M+H-H2O	92736
43.	Isovanillic acid	M+H	349613
44.	Phenyllactic acid	M+H	34529
45.	Phenyllactic acid	M+H	34529
46.	p-Hydroxycinnamic acid	M+H-H2O	354145
47.	Salicylic acid	M+H-H2O	287200
48.	Salicylic acid beta-D-glucoside	M+Na	95685
49.	Salicylic acid, 4-(2-(diethylamino)acetamido)-, hexyl ester, hydrochloride	M+H	427261
50.	Salicylic acid, 4-(3-(diethylamino)propionamido)-, pentyl ester, hydrochloride	M+H-H2O	425741
51.	Salicylic acid, 4-(3-(isopropylamino)propionamido)-, hexyl ester, hydrochloride	M+H	425758
52.	Salicylic acid, 4-amino-, acetate, sodium salt	M+H	392378
53.	Salicylic acid, amino-, 2-hydroxyethyl ester	M+H	428715
54.	Salicylic acid, copper salt	M+NH4	451678
55.	Salicylic acid, methylene ester	M+H-H2	
56.	Salicylic acid, p-butylamino-, 1-ethyl-4-piperidyl ester, hydrochloride	M+H	509254
57.	Stigmasterol	M+H-H2O	168
58.	Vanillic acid	M+H	335542
59.	(R)(-)-Allantoin	M-H	63395
60.	(S)(+)-Allantoin	M-H	63392

61.	2,4-Dihydroxybenzoic acid	M-H	283902
62.	3,4-Dihydroxybenzoic acid	M-H	267460
63.	3',5,7-Trihydroxy-4',8-dimethoxyisoflavone	M-H	302623
64.	3,5-Di-tert-butyl-4-hydroxycinnamic acid, (E)-	M-H	522176
65.	3-Hydroxybenzoic acid	M-H	269175
66.	3-Nitro-4-(piperidin-1-yl)cinnamic acid	M-H	442253
67.	4',5,7-Trihydroxy-3,6-dimethoxyflavone	M-H	455671
68.	4-Hydroxybenzoic acid	M-H	287667
69.	5,7,3'-Trihydroxy-3,4'-dimethoxyflavone 8-C-rhamnopyranoside	M-H	51170
70.	5,7,8-Trihydroxy-3,6,4'-trimethoxy-flavone 8-isovalerate	M-H	51717
71.	Allantoin	M-H	265134
72.	Allantoin	M-H	89
73.	Benzoic acid, 2-(allyloxy)-3,5-dipropyl-, methyl ester	M-H	378753
74.	Benzoic acid, 2,5-dihydroxy-	M-H	337551
75.	Benzoic acid, 2-hydroxy-, compd. with 3-[(2S)-1-methyl-2-pyrrolidinyl]pyridine (1:1)	M-H	386004
76.	Benzoic acid, 3,5-dihydroxy-	M-H	334323
77.	Benzoic acid, 3,5-dimethyl-4-propoxy-, ethyl ester	M-H	387554
78.	Benzoic acid, 3,5-dipropyl-4-((2-methylallyl)oxy)-	M-H	378740
79.	Benzoic acid, 4-(acetyloxy)-3-methoxy-	M-H	398467
80.	Benzoic acid, 4-(allyloxy)-3,5-dipropyl-, methyl ester	M-H	387743
81.	Benzoic acid--[(2R,4R)-2-propyl-1,3-dithiolan-4-yl]methanol (1/1)	M-H	955826
82.	Benzoic acid--[(2R,4S)-2-propyl-1,3-dithiolan-4-yl]methanol (1/1)	M-H	955955
83.	Carbonic acid, ethyl ester, ester with salicylic acid	M-H	426988
84.	Flavone, 5,7-dimethoxy-3,3',4'-trihydroxy-	M-H	421136
85.	Glucosyringic acid	M-H	473754
86.	p-Hydroxycinnamic acid	M-H	354145
87.	Salicylic acid	M-H	287200
88.	Salicylic acid beta-D-glucoside	M-H	95685
89.	Salicylic acid, 3,5-dipropyl-, methyl ester	M-H	378749
90.	Salicylic acid, 4-(N(sup 2)-(1,3-dihydroxy-2-(p-nitrophenyl)-2-propyl)oxamido)-, D-threo-, (-)-	M-H	395938
	Salicylic acid, 4-(N(sup 2)-(1,3-dihydroxy-2-(p-nitrophenyl)-2-propyl)oxamido)-, L-threo-, (+)-	M-H	395941

91.	4-hydroxybenzoic acid- 1-phenylpiperazine(1:1)	M-H	547246
92.	Benzoic acid, 2,2'-[carbonylbis(oxy)]bis-, dimethyl ester	M-H	492589
93.	Benzoic acid, 2,2'-[carbonylbis(oxy)]bis-, dimethyl ester	M-H	492589
94.	5,2',3'-Trihydroxy-7,8-dimethoxyflavone 3'-glucoside	M-H	49717
95.	3,5,6-Trihydroxy-4',7'-dimethoxyflavone	M-H	524365
96.	5,2',6'-Trihydroxy-6,7-dimethoxyflavone 2'-glucoside	M-H	49505
97.	3,5,8-Trihydroxy-7,4'-dimethoxyflavone	M-H	374744

CHEMICALS IN OM 5451			
**No.**	**NAME**	**ADDUCT**	**METLIN ID**
1.	(R)(-)-Allantoin	M+H	63395
2.	(S)(+)-Allantoin	M+H	63392
3.	1-O-p-Coumaroyl-beta-D-glucose	M+NH4	91925
4.	2,3,4-Trihydroxybenzoic acid	M-H	325930
5.	2,4,5-Trihydroxybenzoic acid	M-H	326013
6.	2,4,6-Trihydroxybenzoic acid	M-H	86397
7.	2-Bromo-3-hydroxybenzoic acid	M+H-H2O	852440
8.	2-Bromo-5-hydroxybenzoic acid	M+H-H2O	638875
9.	2-Bromo-6-hydroxybenzoic acid	M+H-H2O	634281
10.	2-Ethyl-6-hydroxybenzoic acid	M+H	276962
11.	2-Hydroxyphenylacetic acid O-b-D-glucoside	M-H	93053
12.	3',5,7-Trihydroxy-4',8-dimethoxyisoflavone	M+Na	302623
13.	3,5,8-Trihydroxy-7,4'-dimethoxyflavone	M+Na	374744
14.	3-Bromo-2-hydroxybenzoic acid	M+H-H2O	340341
15.	3-Bromo-5-hydroxybenzoic acid	M+H-H2O	732273
16.	3-Ethoxy-4-hydroxybenzoic acid	M+H	288553
17.	3-ethyl-2-hydroxybenzoic acid	M+H	300561
18.	3-Hydroxybenzoic acid	M+H-H2O	269175
19.	4-(2,3-Dihydroxypropoxy)-3,5-dihydroxybenzoic acid	M+Na	979387
20.	4,4'-[Hexane-1,6-diylbis(oxy)]bis(2-hydroxybenzoic acid)	M-H	893933
21.	4',5,7-Trihydroxy-3,6-dimethoxyflavone	M+Na	455671
22.	4-Bromo-2-hydroxybenzoic Acid	M+H-H2O	547013
23.	4-Bromo-3-hydroxybenzoic acid	M+H-H2O	580132
24.	4-Hydroxybenzoic acid	M+H-H2O	287667
25.	4-Methoxy-(2E)-cinnamic acid	M+H	313925
26.	5,2',3'-Trihydroxy-7,8-dimethoxyflavone 3'-glucoside	M-H	49717
27.	5,2',6'-Trihydroxy-6,7-dimethoxyflavone 2'-glucoside	M-H	49505
28.	7-Oxostigmasterol	M+H	86666
29.	Allantoic acid	M+H-H2O	343
30.	Allantoin	M+H	265134
31.	Allantoin	M+H	89
32.	Benzoic acid, 2-(allyloxy)-3,5-dipropyl-, methyl ester	M-H	378753

33.	Benzoic acid, 2-(cyanomethyl)-	M+H	357692
34.	Benzoic acid, 2,2'-[carbonylbis(oxy)]bis-, dimethyl ester	M-H	492589
35.	Benzoic acid, 2,4,6-trihydroxy-	M-H	333307
36.	Benzoic acid, 2,4-dihydroxy-3-methyl-, methyl ester	M+H	365361
37.	Benzoic acid, 2,4-dihydroxy-6-methyl-, methyl ester	M+H	347669
38.	Benzoic acid, 2,4-dimethoxy-	M+H	333768
39.	Benzoic acid, 2-bromo-5-fluoro-, methyl ester	M+Na	370122
40.	Benzoic acid, 2-hydroxy-, 2-propenyl ester	M+H-H2O	355726
41.	Benzoic acid, 2-hydroxy-, compd. with 3-[(2S)-1-methyl-2-pyrrolidinyl]pyridine (1:1)	M-H	386004
42.	Benzoic acid, 2-hydroxy-3,4-dimethyl-	M+H	311363
43.	Benzoic acid, 3-(acetyloxy)-4-methoxy-	M-H	322689
44.	Benzoic acid, 3,4-dimethoxy-	M+H	333952
45.	Benzoic acid, 3,5-dimethoxy-	M+H	342868
46.	Benzoic acid, 3,5-dipropyl-4-((2-methylallyl)oxy)-	M-H	378740
47.	Benzoic acid, 3-cyano-, methyl ester	M+H	357246
48.	Benzoic acid, 4-(acetyloxy)-3-methoxy-	M-H	398467
49.	Benzoic acid, 4-(allyloxy)-3,5-dipropyl-, methyl ester	M-H	387743
50.	Benzoic acid, 5-bromo-2-hydroxy-	M+H-H2O	333659
51.	Benzoic acid, acetylmethyl ester	M+H-H2O	358947
52.	Benzoic acid, p-(carboxymethylsulfonyl)-	M+H	524158
53.	Benzoic acid--[(2R,4R)-2-butyl-1,3-dithiolan-4-yl]methanol (1/1)	M-H	955825
54.	Benzoic acid--[(2R,4S)-2-butyl-1,3-dithiolan-4-yl]methanol (1/1)	M-H	955845
55.	beta-Ionone	M+H	269858
56.	Caffeic acid 3-O-glucuronide	M+NH4	96063
57.	Caffeic acid 4-O-glucuronide	M+NH4	96065
58.	Carbonic acid, ethyl ester, ester with salicylic acid	M-H	426988
59.	Cinnamic acid	M+H	386653
60.	Dihydro-beta-ionone	M+H-H2O	317358
61.	Flavone, 4',7-dimethoxy-3,3',5-trihydroxy-	M-H	274757
62.	Flavone, 5,7-dimethoxy-3,3',4'-trihydroxy-	M-H	421136
63.	Homovanillic acid	M+H	336519
64.	Hydrocinnamic acid, p-((2-chloroethyl)(2-fluoroethyl)amino)-	M+NH4	504861
65.	Phenylacetic acid	M+H	269393

66.	Phenyllactic acid	M+H	34529
67.	p-Hydroxycinnamic acid	M+H	354145
68.	Salicylic acid	M+H-H2O	287200
69.	Salicylic acid, 3,5-diallyl-, methyl ester	M+H	519215
70.	Salicylic acid, 4-(2-(diethylamino)acetamido)-, 2-diethylaminoethyl ester, dihydrochloride	M+Na	425764
71.	Salicylic acid, 4-(2-(diethylamino)acetamido)-, pentyl ester, hydrochloride	M-H	425737
72.	Salicylic acid, 4-(3-(diethylamino)propionamido)-, butyl ester, hydrochloride	M-H	425730
73.	Salicylic acid, 4-(3-(isopropylamino)propionamido)-, pentyl ester, hydrochloride	M-H	425743
74.	Salicylic acid, 4-(3-piperidinopropionamido)-, hexyl ester, hydrochloride	M-H	425760
75.	Salicylic acid, 4-(N(sup 2)-(1,3-dihydroxy-2-(p-nitrophenyl)-2-propyl)oxamido)-, D-threo-, (-)-	M+NH4	395938
76.	Salicylic acid, 4-(N(sup 2)-(1,3-dihydroxy-2-(p-nitrophenyl)-2-propyl)oxamido)-, L-threo-, (+)-	M+NH4	395941
77.	Salicylic acid, amino-, 2-hydroxyethyl ester	M+H	428715
78.	trans-o-Coumaric acid 2-glucoside	M+NH4	89390
79.	trans-p-Coumaric acid 4-glucoside	M+NH4	94078
80.	Vanillic acid 4-sulfate	M+H	96140

CHEMICALS IN OM 6976			
**No.**	**NAME**	**ADDUCT**	**METLIN ID**
1.	(R)(-)-Allantoin	M+H	63395
2.	(S)(+)-Allantoin	M+H	63392
3.	1-(2-Chloro-4-nitroanilino)-5-methoxyanthracene-9,10-dione	M-H	754860
4.	1-Acetyl-2,4,5,7-tetramethoxyanthracene-9,10-dione	M+H	648775
5.	2,2'-(Butane-1,4-diyldiazanediyl)bis(phenylacetic acid)--hydrogen bromide (1/1)	M-H	839118
6.	2,2'-(Decane-1,10-diyldiazanediyl)bis(phenylacetic acid)--hydrogen bromide (1/1)	M-H	841096
7.	2,2'-(Propane-1,3-diyldiazanediyl)bis(phenylacetic acid)--hydrogen bromide (1/1)	M-H	840611
8.	2,4-Dihydroxybenzaldehyde	M-H	270019
9.	2,4-Dihydroxybenzoic acid	M-H	283902
10.	2,5-Bis(trifluoromethyl)hydrocinnamic acid	M+H	492329
11.	2,5-Dihydroxybenzoic Acid-d3	M-H	602156
12.	2-bromo-2,2-diphenylacetic acid	M-H	283530
13.	2-Bromo-3-hydroxybenzoic acid	M+H-H2O	852440
14.	2-Bromo-5-hydroxybenzoic acid	M+H-H2O	638875
15.	2-Bromo-6-hydroxybenzoic acid	M+H-H2O	634281
16.	2-Ethyl-6-hydroxybenzoic acid	M+H	276962
17.	2-Hydroxyphenylacetic acid O-b-D-glucoside	M-H	93053
18.	3-(Trifluoromethyl)benzoic acid	M+Na	303381
19.	3-(trifluoromethyl)-Benzoic acid	M+Na	1959
20.	3,4-Dihydroxybenzoic acid	M-H	267460
21.	3,5-Bis(trifluoromethyl)hydrocinnamic acid	M+H	419911
22.	3,5-Di-tert-butyl-4-hydroxyhydocinnamic acid	M-H	361036
23.	3-{[Bis(carboxymethyl)amino]methyl}-2-hydroxybenzoic acid	M+Na	694777
24.	3-Bromo-2-hydroxybenzoic acid	M+H-H2O	340341
25.	3-Bromo-5-hydroxybenzoic acid	M+H-H2O	732273
26.	3-bromo-5-phenyl Salicylic Acid	M+H	64822
27.	3-ethyl-2-hydroxybenzoic acid	M+H	300561
28.	3-Hexadecyl-2-hydroxybenzoic acid	M-H	758916
29.	3-Hexaprenyl-4,5-Dihydroxybenzoic acid	M+NH4	5976

30.	3-hexaprenyl-4-hydroxybenzoic acid	M-H	58554
31.	3-Hydroxybenzoic acid	M+H-H2O	269175
32.	3-Nitro-4-(piperidin-1-yl)cinnamic acid	M-H	442253
33.	4-Bromo-2-hydroxybenzoic Acid	M+H-H2O	547013
34.	4-Bromo-3-hydroxybenzoic acid	M+H-H2O	580132
35.	4-Formyl-2-hydroxybenzoic acid	M+H-H2O	392342
36.	4-Formyl-3-hydroxybenzoic acid	M+H-H2O	562697
37.	4-Hydroxybenzoic acid	M+H-H2O	287667
38.	4-tert-Butyl-2-hydroxybenzoic acid--copper (2/1)	M+H-H2O	784133
39.	5-bromo-3-pheny Salicylic Acid	M+H	64931
40.	8-Quinolinol salicylic acid (1:1)	M-H	311563
41.	9-Hydroxy-7-megastigmen-3-one glucoside	M+Na	95175
42.	Allantoic acid	M+H-H2O	343
43.	Allantoin	M+H	265134
44.	Allantoin	M+H	89
45.	Benzoic acid, 2-(allyloxy)-3,5-dipropyl-, methyl ester	M-H	378753
46.	Benzoic acid, 2,2'-[1,4-phenylenebis(carbonylimino)]bis[5-hydroxy-	M+H	493792
47.	Benzoic acid, 2,5-dihydroxy-	M-H	337551
48.	Benzoic acid, 2-[(1,3-benzodioxol-5-ylmethylene)amino]-, methyl ester	M-H	378871
49.	Benzoic acid, 2-[(carboxymethyl)amino]-, monosodium salt	M+H	374048
50.	Benzoic acid, 2-decyl-6-hydroxy-	M-H	587378
51.	Benzoic acid, 2-hydroxy-, 2-propenyl ester	M+H	355726
52.	Benzoic acid, 2-hydroxy-3,4-dimethyl-	M+H	311363
53.	Benzoic acid, 2-hydroxy-6-pentadecyl-, methyl ester	M-H	598138
54.	Benzoic acid, 3,5-bis(1,1-dimethylethyl)-4-hydroxy-, ethyl ester	M-H	343827
55.	Benzoic acid, 3,5-dihydroxy-	M-H	334323
56.	Benzoic acid, 3,5-dimethyl-4-propoxy-, et		
57.	Benzoic acid, 3,5-dipropyl-4-((2-methylallyl)oxy)-	M-H	378740
58.	Benzoic acid, 3,5-dipropyl-4-ethoxy-, ethyl ester	M-H	388040
59.	Benzoic acid, 3-hydroxy-5-methyl-	M+H	313087
60.	Benzoic acid, 4-(allyloxy)-3,5-dipropyl-, methyl ester	M-H	387743
61.	Benzoic acid, 4-(trifluoromethyl)-	M+Na	337338
62.	Benzoic acid, 5-bromo-2-hydroxy-	M+H-H2O	333659

63.	Benzoic acid, 5-hexadecyl-2-hydroxy-	M-H	364044
64.	Benzoic acid, p-hydroxy-, decyl ester	M-H	371436
65.	Benzoic acid--[(2R,4R)-2-butyl-1,3-dithiolan-4-yl]methanol (1/1)	M-H	955825
66.	Benzoic acid--[(2R,4R)-2-propyl-1,3-dithiolan-4-yl]methanol (1/1)	M-H	955826
67.	Benzoic acid--[(2R,4S)-2-butyl-1,3-dithiolan-4-yl]methanol (1/1)	M-H	955845
68.	Benzoic acid--[(2R,4S)-2-propyl-1,3-dithiolan-4-yl]methanol (1/1)	M-H	955955
69.	Benzoic acid--benzene-1,3,5-triol (2/1)	M+H	929628
70.	Caffeic acid 3-O-glucuronide	M+NH4	96063
71.	Caffeic acid 4-O-glucuronide	M+NH4	96065
72.	Cinnamic acid	M+H	386653
73.	Dihydro-beta-ionone	M+H-H2O	317358
74.	Dihydroferulic acid 4-O-glucuronide	M-H	96081
75.	Dimethyl 2,3,6,7-tetramethoxyanthracene-9,10-dicarboxylate	M+H	881654
76.	Hydrocinnamic acid, p-((2-chloroethyl)(2-fluoroethyl)amino)-	M+NH4	504861
77.	Phenylacetic acid	M+H	269393
78.	p-Hydroxycinnamic acid	M+H	354145
79.	Salicylic acid	M+H-H2O	287200
80.	Salicylic acid beta-D-glucoside	M-H	95685
81.	Salicylic acid, 3,5-dipropyl-, methyl ester	M-H	378749
82.	Salicylic acid, 4-(2-(diethylamino)acetamido)-, hexyl ester, hydrochloride	M+H	427261
83.	Salicylic acid, 4-(2-piperidinoacetamido)-, butyl ester, hydrochloride	M+H-H2O	425724
84.	Salicylic acid, 4-(2-piperidinoacetamido)-, hexyl ester, hydrochloride	M+NH4	425754
85.	Salicylic acid, 4-(3-(cyclohexylamino)propionamido)-, ethyl ester, monohydrochloride	M+H-H2O	425715
86.	Salicylic acid, 4-(3-(diethylamino)propionamido)-, pentyl ester, hydrochloride	M+H	425741
87.	Salicylic acid, 4-(3-(isopropylamino)propionamido)-, hexyl ester, hydrochloride	M+H	425758
88.	Salicylic acid, 4-(3-piperidinopropionamido)-, pentyl ester, hydrochloride	M+NH4	425745
89.	Salicylic acid, 4-(3-piperidinopropionamido)-, propyl ester, hydrochloride	M+H-H2O	425719

90.	Salicylic acid, 4-(4-oxo-4H-1-benzopyran-2-carboxamido)-	M+H	472951
91.	Salicylic acid, 4-amino-, acetate, sodium salt		392378
92.	Salicylic acid, p-butylamino-, 1-ethyl-4-piperidyl ester, hydrochloride	M+H	509254
93.	Syringic acid-copper(II) complex	M+NH4	351587
94.	trans-o-Coumaric acid 2-glucoside	M+NH4	89390
95.	trans-p-Coumaric acid 4-glucoside	M+NH4	94078

**CHEMICALS IN OM 7347**			
**No.**	**NAME**	**ADDUCT**	**METLIN ID**
1.	2,4-Dihydroxy-6-(2-oxopropyl)benzoic acid	M-H	410207
2.	2-amino-3,5-dihydroxybenzoic acid	M+H	330220
3.	2-Ethyl-6-hydroxybenzoic acid	M+H-H2O	276962
4.	3,4-Difluorohydrocinnamic acid	M+H-H2O	462174
5.	3',5,7-Trihydroxy-4',8-dimethoxyisoflavone	M+H	302623
6.	3,5,8-Trihydroxy-7,4'-dimethoxyflavone	M+H	374744
7.	3,5-Di-tert-butyl-4-hydroxycinnamic acid, (E)-	M+H	522176
8.	3-ethyl-2-hydroxybenzoic acid	M+H-H2O	300561
9.	3-Hexaprenyl-4,5-Dihydroxybenzoic acid	M+NH4	5976
10.	3-hexaprenyl-4-hydroxybenzoic acid	M-H	58554
11.	3-Nitro-4-(piperidin-1-yl)cinnamic acid	M-H	442253
12.	4-(2,3-Dihydroxypropoxy)-3,5-dihydroxybenzoic acid	M-H	979387
13.	4-(decanoylamino)-2-hydroxybenzoic acid	M+NH4	483818
14.	4,4'-[Hexane-1,6-diylbis(oxy)]bis(2-hydroxybenzoic acid)	M-H	893933
15.	4,4'-[Propane-1,3-diylbis(oxy)]bis(2-hydroxybenzoic acid)	M+H-H2O	893932
16.	4-amino-2,6-dihydroxybenzoic acid	M+H	534515
17.	4-Formyl-2-hydroxybenzoic acid	M+H-H2O	392342
18.	4-Formyl-3-hydroxybenzoic acid	M+H-H2O	562697
19.	5-Amino-2,4-dihydroxybenzoic acid	M+H	288841
20.	7-Oxostigmasterol	M+H	86666
21.	Benzoic acid, 2-(allyloxy)-3,5-dipropyl-, methyl ester	M-H	378753
22.	Benzoic acid, 2-bromo-5-fluoro-, methyl ester	M+Na	370122
23.	Benzoic acid, 2-hydroxy-3,4-dimethyl-	M+H-H2O	311363
24.	Benzoic acid, 2-hydroxy-3-iodo-	M+H	643590

25.	Benzoic acid, 3-(acetyloxy)-4-methoxy-	M-H	322689
26.	Benzoic acid, 3,5-dipropyl-4-((2-methylallyl)oxy)-	M+H	378740
27.	Benzoic acid, 3-iodosyl-	M+H	287937
28.	Benzoic acid, 4-(acetyloxy)-3-methoxy-	M-H	398467
29.	Benzoic acid, 4-(allyloxy)-3,5-dipropyl-, methyl ester	M+H	387743
30.	Benzoic acid, 5-formyl-2-(2-methoxy-2-oxoethoxy)-, methyl ester	M-H	576670
31.	Benzoic acid, p-((2-((2-mercaptoethyl)amino)ethyl)amino)-, ethyl ester, hydrogen sulfate (ester)	M-H	457419
32.	Benzoic acid--[(2R,4R)-2-propyl-1,3-dithiolan-4-yl]methanol (1/1)	M-H	955826
33.	Benzoic acid--[(2R,4S)-2-propyl-1,3-dithiolan-4-yl]methanol (1/1)	M-H	955955
34.	Carbonic acid, ethyl ester, ester with salicylic acid	M-H	426988
35.	Dihydroferulic acid 4-O-glucuronide	M-H	96081
36.	Flavone, 4',7-dimethoxy-3,3',5-trihydroxy-	M+H	274757
37.	Flavone, 5,7-dimethoxy-3,3',4'-trihydroxy-	M+H	421136
38.	Hydrocinnamic acid, p-((2-chloroethyl)(2-fluoroethyl)amino)-	M+NH4	504861
39.	Phenylacetic acid	M+H-H2O	269393
40.	p-Hydroxycinnamic acid	M-H	354145
41.	Salicylic acid beta-D-glucoside	M-H	95685
42.	Salicylic acid, 4-(2-(diethylamino)acetamido)-, propyl ester, hydrochloride	M+NH4	425716
43.	Salicylic acid, 4-(2-piperidinoacetamido)-, butyl ester, hydrochloride	M+H-H2O	425724
44.	Salicylic acid, 4-(3-(cyclohexylamino)propionamido)-, ethyl ester, monohydrochloride	M+H-H2O	425715
45.	Salicylic acid, 4-(3-(diethylamino)propionamido)-, ethyl ester, hydrochloride	M+NH4	425711
46.	Salicylic acid, 4-(3-(isopropylamino)propionamido)-, propyl ester, hydrochloride	M+NH4	425977
47.	Salicylic acid, 4-(3-piperidinopropionamido)-, propyl ester, hydrochloride	M+H-H2O	425719
48.	Salicylic acid, copper salt	M+NH4	451678
49.	Salicylic acid, p-butylamino-, 1-ethyl-4-piperidyl ester, hydrochloride	M+H	509254

CHEMICALS IN OM N406			
**No.**	**NAME**	**ADDUCT**	**METLIN ID**
1.	(R)(-)-Allantoin	M+H	63395
2.	(S)(+)-Allantoin	M+H	63392
3.	{4-[(Aminooxy)methyl]phenyl}acetic acid--hydrogen bromide (1/1)	M+H-H2O	818607
4.	2,2'-(Propane-1,3-diyldiazanediyl)bis(phenylacetic acid)--hydrogen bromide (1/1)	M+H	840611
5.	2,4-Dihydroxybenzoic acid	M+H-H2O	283902
6.	2-Ethyl-6-hydroxybenzoic acid	M+H	276962
7.	3-(2-Aminoethyl)-2-hydroxybenzoic acid--hydrogen bromide (1/1)	M+H-H2O	982609
8.	3,4-Dihydroxybenzoic acid	M+H-H2O	267460
9.	3',5,7-Trihydroxy-4',8-dimethoxyisoflavone	M+H	302623
10.	3,5,8-Trihydroxy-7,4'-dimethoxyflavone	M+H	374744
11.	3,5-Di-tert-butyl-4-hydroxycinnamic acid, (E)-	M+H	522176
12.	3-[(tert-Butoxycarbonyl)amino]-5-hydroxybenzoic acid	M+Na	286137
13.	3-Ethoxy-4-hydroxybenzoic acid	M+H	288553
14.	3-ethyl-2-hydroxybenzoic acid	M+H	300561
15.	3-Hexadecyl-2-hydroxybenzoic acid	M+NH4	758916
16.	4-(decanoylamino)-2-hydroxybenzoic acid	M+NH4	483818
17.	4,4'-[Propane-1,3-diylbis(oxy)]bis(2-hydroxybenzoic acid)	M+H-H2O	893932
18.	4-amino-2-hydroxybenzoic acid- 10-[2-(pyrrolidin-1-yl)ethyl]-10h-phenothiazine(1:1)	M+Na	528266
19.	4-Methoxy-(2E)-cinnamic acid	M+H-H2O	313925
20.	7-Oxostigmasterol	M+H-H2O	86666
21.	Allantoic acid	M+H-H2O	343
22.	Allantoin	M+H	265134
23.	Allantoin	M+H	89
24.	Benzoic acid--(1R,2S)-cyclohexa-3,5-diene-1,2-diol (2/1)	M+H	952179
25.	Benzoic acid, 2-(allyloxy)-3,5-dipropyl-, methyl ester	M+H	378753
26.	Benzoic acid, 2-(cyanomethyl)-	M+H	357692
27.	Benzoic acid, 2,2'-[1,4-phenylenebis(carbonylimino)]bis-	M+H	416785
28.	Benzoic acid, 2,4-dihydroxy-3-methyl-, methyl ester	M+H	365361

29.	Benzoic acid, 2,4-dihydroxy-6-methyl-, methyl ester	M+H	347669
30.	Benzoic acid, 2,4-dimethoxy-	M+H	333768
31.	Benzoic acid, 2,5-dihydroxy-	M+H-H2O	337551
32.	Benzoic acid, 2-[(carboxymethyl)amino]-, monosodium salt	M+H	374048
33.	Benzoic acid, 2-hydroxy-6-pentadecyl-, methyl ester	M+NH4	598138
34.	Benzoic acid, 3-(4-carboxyphenoxy)-4-methoxy-	M+H-H2O	386733
35.	Benzoic acid, 3,3'-[1,3-phenylenebis(carbonylimino)]bis-	M+H	364552
36.	Benzoic acid, 3,4-dimethoxy-	M+H	333952
37.	Benzoic acid, 3,5-dihydroxy-	M+H-H2O	334323
38.	Benzoic acid, 3,5-dimethoxy-	M+H	342868
39.	Benzoic acid, 3,5-dipropyl-4-((2-methylallyl)oxy)-	M+H	378740
40.	Benzoic acid, 3-cyano-, methyl ester	M+H	357246
41.	Benzoic acid, 4-(allyloxy)-3,5-dipropyl-, methyl ester	M+H	387743
42.	Benzoic acid, 5-formyl-2-(2-methoxy-2-oxoethoxy)-, methyl ester	M+H	576670
43.	Benzoic acid, 5-hexadecyl-2-hydroxy-	M+NH4	364044
44.	beta-Ionone	M+H	269858
45.	Caffeic acid 3-O-glucuronide	M+NH4	96063
46.	Caffeic acid 4-O-glucuronide	M+NH4	96065
47.	Cinnamic acid	M+H	386653
48.	cis-Ferulic acid [arabinosyl-(1->3)-[glucosyl-(1->6)]-glucosyl] ester	M+H	90133
49.	Dihydro-beta-ionone	M+H	317358
50.	Dihydrocaffeic acid 3-O-glucuronide	M+Na	96078
51.	Dihydroferulic acid 4-O-glucuronide	M+Na	96081
52.	E-Cinnamic acid	M+H	273681
53.	Flavone, 4',7-dimethoxy-3,3',5-trihydroxy-	M+H	274757
54.	Flavone, 5,7-dimethoxy-3,3',4'-trihydroxy-	M+H	421136
55.	Homovanillic acid	M+H	336519
56.	Hydrocinnamic acid, p-((2-chloroethyl)(2-fluoroethyl)amino)-	M+NH4	504861
57.	Phenylacetic acid	M+H	269393
58.	p-Hydroxycinnamic acid	M+H-H2O	354145
59.	Salicylic acid, 4-(2-(diethylamino)acetamido)-, ethyl ester, hydrochloride	M+H	417446
60.	Salicylic acid, 4-(2-(diethylamino)acetamido)-, hexyl ester, hydrochloride	M+H	427261

61.	Salicylic acid, 4-(3-(diethylamino)propionamido)-, methyl ester, hydrochloride	M+H	426173
62.	Salicylic acid, 4-(3-(diethylamino)propionamido)-, pentyl ester, hydrochloride	M+H	425741
63.	Salicylic acid, 4-(3-(isopropylamino)propionamido)-, ethyl ester, hydrochloride	M+H	417199
64.	Salicylic acid, 4-(3-(isopropylamino)propionamido)-, hexyl ester, hydrochloride	M+H	425758
65.	Salicylic acid, 4-(N(sup 2)-(1,3-dihydroxy-2-(p-nitrophenyl)-2-propyl)oxamido)-, D-threo-, (-)-	M+NH4	395938
66.	Salicylic acid, 4-(N(sup 2)-(1,3-dihydroxy-2-(p-nitrophenyl)-2-propyl)oxamido)-, L-threo-, (+)-	M+NH4	395941
67.	Salicylic acid, 4-amino-, acetate, sodium salt	M+H	392378
68.	Salicylic acid, amino-, 2-hydroxyethyl ester	M+H	428715
69.	Salicylic acid, copper salt	M+NH4	451678
70.	Salicylic acid, methylene ester	M+H-H2O	506200
71.	Salicylic acid, p-amino-, 1-methyl-4-piperidyl ester, hydrochloride	M+H	509235
72.	Salicylic acid, p-butylamino-, 1-ethyl-4-piperidyl ester, hydrochloride	M+H	509254
73.	2-(Hexadec-9-en-1-yl)-6-hydroxybenzoic acid	M-H	911630
74.	2,6-Dimethoxybenzoic acid	M-H	316720
75.	2-Ethyl-6-hydroxybenzoic acid	M-H	276962
76.	2-Hydroxyphenylacetic acid O-b-D-glucoside	M-H	93053
77.	3,5-Di-tert-butyl-4-hydroxycinnamic acid, (E)-	M-H	522176
78.	3,5-Di-tert-butyl-4-hydroxyhydocinnamic acid	M-H	361036
79.	3-Ethoxy-4-hydroxybenzoic acid	M-H	288553
80.	3-ethyl-2-hydroxybenzoic acid	M-H	300561
81.	3-hexaprenyl-4-hydroxybenzoic acid	M-H	58554
82.	3-Hydroxybenzoic acid	M-H	269175
83.	4-Hydroxybenzoic acid	M-H	287667
84.	4-hydroxybenzoic acid- 1-phenylpiperazine(1:1)	M-H	547246
85.	8-Quinolinol salicylic acid (1:1)	M-H	311563
86.	Benzoic acid, 2-(allyloxy)-3,5-dipropyl-, methyl ester	M-H	378753
87.	Benzoic acid, 2,4-dihydroxy-3-methyl-, methyl ester	M-H	365361
88.	Benzoic acid, 2,4-dihydroxy-6-methyl-, methyl ester	M-H	347669
89.	Benzoic acid, 2,4-dimethoxy-	M-H	333768

90.	Benzoic acid, 2-[(1,3-benzodioxol-5-ylmethylene)amino]-, methyl ester	M-H	378871
91.	Benzoic acid, 2-decyl-6-hydroxy-	M-H	587378
92.	Benzoic acid, 2-hydroxy-, compd. with 3-[(2S)-1-methyl-2-pyrrolidinyl]pyridine (1:1)	M-H	386004
93.	Benzoic acid, 2-hydroxy-3,4-dimethyl-	M-H	311363
94.	Benzoic acid, 3-(acetyloxy)-4-methoxy-	M-H	322689
95.	Benzoic acid, 3,4-dimethoxy-	M-H	333952
96.	Benzoic acid, 3,5-bis(1,1-dimethylethyl)-4-hydroxy-, ethyl ester	M-H	343827
97.	Benzoic acid, 3,5-bis(acetyloxy)-4-(phenylmethoxy)-, methyl ester	M-H	601090
98.	Benzoic acid, 3,5-dimethoxy-	M-H	342868
99.	Benzoic acid, 3,5-dimethyl-4-propoxy-, ethyl ester	M-H	387554
100.	Benzoic acid, 3,5-dipropyl-4-((2-methylallyl)oxy)-	M-H	378740
101.	Benzoic acid, 3,5-dipropyl-4-ethoxy-, ethyl ester	M-H	388040
102.	Benzoic acid, 4-(acetyloxy)-3-methoxy-	M-H	398467
103.	Benzoic acid, 4-(allyloxy)-3,5-dipropyl-, methyl ester	M-H	387743
104.	Benzoic acid, p-hydroxy-, decyl ester	M-H	371436
105.	Benzoic acid--[(2R,4R)-2-butyl-1,3-dithiolan-4-yl]methanol (1/1)	M-H	955825
106.	Benzoic acid--[(2R,4R)-2-propyl-1,3-dithiolan-4-yl]methanol (1/1)	M-H	955826
107.	Benzoic acid--[(2R,4S)-2-butyl-1,3-dithiolan-4-yl]methanol (1/1)	M-H	955845
108.	Benzoic acid--[(2R,4S)-2-propyl-1,3-dithiolan-4-yl]methanol (1/1)	M-H	955955
109.	Carbonic acid, ethyl ester, ester with salicylic acid	M-H	426988
110.	Dihydroferulic acid 4-O-glucuronide	M-H	96081
111.	Homovanillic acid	M-H	336519
112.	Hydrocinnamic acid, m-(bis(2-fluoroethyl)amino)-	M-H	406340
113.	Hydrocinnamic acid, p-(bis(2-fluoroethyl)amino)-	M-H	503631
114.	p-Hydroxycinnamic acid	M-H	354145
115.	Salicylic acid	M-H	287200
116.	Salicylic acid beta-D-glucoside	M-H	95685
117.	Salicylic acid, 3,5-dipropyl-, methyl ester	M-H	378749
118.	Salicylic acid, 4-(3-piperidinopropionamido)-, hexyl ester, hydrochloride	M-H	425760
119.	Salicylic acid, heptanoate	M-H	378999

CHEMICALS IN OM 380			
No.	NAME	ADDUCT	METLIN ID
1.	(R)(-)-Allantoin	M+H	63395
2.	(S)(+)-Allantoin	M+H	63392
3.	2-(Hexadec-9-en-1-yl)-6-hydroxybenzoic acid	M-H	911630
4.	2,2'-(Decane-1,10-diyldiazanediyl)bis(phenylacetic acid)--hydrogen bromide (1/1)	M-H	841096
5.	2,4-Dihydroxy-6-(2-oxopropyl)benzoic acid	M-H	410207
6.	2,6-Dimethoxybenzoic acid	M+H	316720
7.	2-bromo-2,2-diphenylacetic acid	M-H	283530
8.	2-Bromo-3-hydroxybenzoic acid	M+H-H2O	852440
9.	2-Bromo-5-hydroxybenzoic acid	M+H-H2O	638875
10.	2-Bromo-6-hydroxybenzoic acid	M+H-H2O	634281
11.	2-Ethyl-6-hydroxybenzoic acid	M+H	276962
12.	2-hydroxybenzoic acid- 5-(4-chlorophenyl)-6-ethylpyrimidine-2,4-diamine(1:1)	M+H-H2O	535077
13.	2-Hydroxyphenylacetic acid O-b-D-glucoside	M-H	93053
14.	3,5,8-Trihydroxy-7,4'-dimethoxyflavone	M+H	374744
15.	3,5-Di-tert-butyl-4-hydroxycinnamic acid, (E)-	M-H	522176
16.	3,5-Di-tert-butyl-4-hydroxyhydocinnamic acid	M-H	361036
17.	3-Bromo-2-hydroxybenzoic acid	M+H-H2O	340341
18.	3-Bromo-5-hydroxybenzoic acid	M+H-H2O	732273
19.	3-Ethoxy-4-hydroxybenzoic acid	M+H	288553
20.	3-ethyl-2-hydroxybenzoic acid	M+H	300561
21.	3-Hexadecyl-2-hydroxybenzoic acid	M+NH4	758916
22.	3-Hexaprenyl-4,5-Dihydroxybenzoic acid	M-H	5976
23.	3-hexaprenyl-4-hydroxybenzoic acid	M-H	58554
24.	3-Nitro-4-(piperidin-1-yl)cinnamic acid	M-H	442253
25.	4-(decanoylamino)-2-hydroxybenzoic acid	M+NH4	483818
26.	4,4'-[Propane-1,3-diylbis(oxy)]bis(2-hydroxybenzoic acid)	M+H-H2O	893932
27.	4-[(E)-{[4-(Bis{2-[(methanesulfonyl)oxy]ethyl}amino)phenyl]methylidene}amino]-2-hydroxybenzoic acid--hydrogen chloride (1/1)	M-H	787376
28.	4-Bromo-2-hydroxybenzoic Acid	M+H-H2O	547013
29.	4-Bromo-3-hydroxybenzoic acid	M+H-H2O	580132
30.	7-Oxostigmasterol	M+H-H2O	86666
31.	8-Quinolinol salicylic acid (1:1)	M-H	311563
32.	9-Hydroxy-7-megastigmen-3-one glucoside	M+Na	95175
33.	Allantoin	M+H	265134
34.	Allantoin	M+H	89

35.	Benzoic acid, 2-(allyloxy)-3,5-dipropyl-, methyl ester	M-H	378753
36.	Benzoic acid, 2,2'-[1,4-phenylenebis(carbonylimino)]bis[5-hydroxy-	M+H	493792
37.	Benzoic acid, 2,4-dihydroxy-6-methyl-, methyl ester	M+H	347669
38.	Benzoic acid, 2,4-dimethoxy-	M+H	333768
39.	Benzoic acid, 2-[(1,3-benzodioxol-5-ylmethylene)amino]-, methyl ester	M-H	378871
40.	Benzoic acid, 2-hydroxy-, 2-propenyl ester	M+H-H2O	355726
41.	Benzoic acid, 2-hydroxy-3,4-dimethyl-	M+H	311363
42.	Benzoic acid, 2-hydroxy-6-pentadecyl-, methyl ester	M+NH4	598138
43.	Benzoic acid, 3-(acetyloxy)-4-methoxy-	M-H	322689
44.	Benzoic acid, 3,4-dimethoxy-	M+H	333952
45.	Benzoic acid, 3,5-bis(1,1-dimethylethyl)-4-hydroxy-, ethyl ester	M-H	343827
46.	Benzoic acid, 3,5-dimethoxy-	M+H	342868
47.	Benzoic acid, 3,5-dimethyl-4-propoxy-, ethyl ester	M-H	387554
48.	Benzoic acid, 3,5-dipropyl-4-((2-methylallyl)oxy)-	M-H	378740
49.	Benzoic acid, 3,5-dipropyl-4-ethoxy-, ethyl ester	M-H	388040
50.	Benzoic acid, 3-cyano-, methyl ester	M+H	357246
51.	Benzoic acid, 4-(acetyloxy)-3-methoxy-	M-H	398467
52.	Benzoic acid, 4-(allyloxy)-3,5-dipropyl-, methyl ester	M-H	387743
53.	Benzoic acid, 5-bromo-2-hydroxy-	M+H-H2O	333659
54.	Benzoic acid, 5-hexadecyl-2-hydroxy-	M+NH4	364044
55.	Benzoic acid, acetylmethyl ester	M+H-H2O	358947
56.	Benzoic acid, p-hydroxy-, decyl ester	M-H	371436
57.	Benzoic acid--[(2R,4R)-2-butyl-1,3-dithiolan-4-yl]methanol (1/1)	M-H	955825
58.	Benzoic acid--[(2R,4R)-2-propyl-1,3-dithiolan-4-yl]methanol (1/1)	M-H	955826
59.	Benzoic acid--[(2R,4S)-2-butyl-1,3-dithiolan-4-yl]methanol (1/1)	M-H	955845
60.	Benzoic acid--[(2R,4S)-2-propyl-1,3-dithiolan-4-yl]methanol (1/1)	M-H	955955
61.	beta-Ionone	M+H	269858
62.	Caffeic acid 3-O-glucuronide	M+NH4	96063
63.	Caffeic acid 4-O-glucuronide	M+NH4	96065
64.	Carbonic acid, ethyl ester, ester with salicylic acid	M-H	426988
65.	Cinnamic acid	M+H	386653

66.	Dihydroferulic acid 4-O-glucuronide	M-H	96081
67.	E-Cinnamic acid	M+H	273681
68.	Flavone, 4',7-dimethoxy-3,3',5-trihydroxy-	M+H	274757
69.	Flavone, 5,7-dimethoxy-3,3',4'-trihydroxy-	M+H	421136
70.	Homovanillic acid	M+H	336519
71.	Hydrocinnamic acid, p-((2-chloroethyl)(2-fluoroethyl)amino)-	M+NH4	504861
72.	Phenylacetic acid	M+H-H2O	269393
73.	p-Hydroxycinnamic acid	M+H-H2O	354145
74.	Salicylic acid beta-D-glucoside	M-H	95685
75.	Salicylic acid, 3,5-diallyl-, methyl ester	M+H	519215
76.	Salicylic acid, 3,5-dipropyl-, methyl ester	M-H	378749
77.	Salicylic acid, 4-(2-piperidinoacetamido)-, butyl ester, hydrochloride	M+H-H2O	425724
78.	Salicylic acid, 4-(3-(cyclohexylamino)propionamido)-, ethyl ester, monohydrochloride	M+H-H2O	425715
79.	Salicylic acid, 4-(3-moprholinopropionamido)-, butyl ester, hydrochloride	M+H	425735
80.	Salicylic acid, 4-(3-piperidinopropionamido)-, propyl ester, hydrochloride	M+H-H2O	425719
81.	Salicylic acid, p-amino-, 1-methyl-4-piperidyl ester, hydrochloride	M+H	509235
82.	Salicylic acid, p-butylamino-, 1-ethyl-4-piperidyl ester, hydrochloride	M+H	509254

*: The identification level is 2.
